# The evolution and population structure of *Lactobacillus fermentum* from different naturally fermented products as determined by multilocus sequence typing (MLST)

**DOI:** 10.1186/s12866-015-0447-z

**Published:** 2015-05-20

**Authors:** Tong Dan, Wenjun Liu, Yuqin Song, Haiyan Xu, Bilige Menghe, Heping Zhang, Zhihong Sun

**Affiliations:** Key Laboratory of Dairy Biotechnology and Engineering, Education Ministry of P. R. China, Department of Food Science and Engineering, Inner Mongolia Agricultural University, Hohhot, 010018 P. R. China

**Keywords:** *Lactobacillus fermentum*, multilocus sequence typing (MLST), Population structure, Traditional Fermented food, Sequence type (ST), Housekeeping gene

## Abstract

**Background:**

*Lactobacillus fermentum* is economically important in the production and preservation of fermented foods. A repeatable and discriminative typing method was devised to characterize *L. fermentum* at the molecular level. The multilocus sequence typing (MLST) scheme developed was based on analysis of the internal sequence of 11 housekeeping gene fragments (*clpX*, *dnaA*, *dnaK*, *groEL*, *murC, murE*, *pepX*, *pyrG*, *recA*, *rpoB*, and *uvrC*).

**Results:**

MLST analysis of 203 isolates of *L. fermentum* from Mongolia and seven provinces/ autonomous regions in China identified 57 sequence types (ST), 27 of which were represented by only a single isolate, indicating high genetic diversity. Phylogenetic analyses based on the sequence of the 11 housekeeping gene fragments indicated that the *L. fermentum* isolates analyzed belonged to two major groups. A standardized index of association (*I*_*A*_^S^) indicated a weak clonal population structure in *L. fermentum*. Split decomposition analysis indicated that recombination played an important role in generating the genetic diversity observed in *L. fermentum*. The results from the minimum spanning tree strongly suggested that evolution of *L. fermentum* STs was not correlated with geography or food-type.

**Conclusions:**

The MLST scheme developed will be valuable for further studies on the evolution and population structure of *L. fermentum* isolates used in food products.

**Electronic supplementary material:**

The online version of this article (doi:10.1186/s12866-015-0447-z) contains supplementary material, which is available to authorized users.

## Background

*Lactobacillus fermentum* is an economically important species of lactic acid bacterium (LAB) used in the production and preservation of fermented food as an acid-producing starter culture [1, 2]. Isolates of *L. fermentum* originate from a variety of habitats: traditionally fermented milk products, sourdough, fermenting plant materials, faeces and sewage amongst others [3–8]. *Lactobacillus fermentum* was first described by Beijerink (1901), as an obligate heterofermentative bacterium associated with the fermentation of hexoses to lactic acid [9, 10]. Another species, *Lactobacillus cellobiosus*, which was first described by Rogosa et al., (1953) is also heterofermentative and DNA-DNA hybridization studies showed it to be very similar to *L. fermentum* [11, 12]. There is now strong evidence to support a close relationship between *L. cellobiosus* and *L. fermentum* and, in fact, *L. cellobiosus* has now been reclassified as a biovar of *L. fermentum* [5, 13].

In recent years, molecular typing approaches have been used to characterize *L. fermentum* and the subspecies within it. For example, *L. fermentum* isolates could be differentiated from other *Lactobacillus* species using randomly amplified polymorphic DNA(RAPD-PCR) methods [14, 15]. RAPD-PCR has also been used in combination with amplified 16S rDNA restriction analysis (16S-ARDRA), pulsed-field gel electrophoresis with restriction fragment length polymorphism (PFGE-RFLP) and ribotyping to characterize 178 isolates from *Lactobacillus* species in wine [16]. More recently, *L. fermentum* has been differentiated from *L. gasseri* and *L. plantarum* isolates from the human genital tract using PFGE and fluorescence in situ hybridization (FISH) [17]. However, these methods can sometimes be ambiguous because the majority of bacteria have very similar nutritional requirements and grow under similar environmental conditions [18].

Multilocus sequence typing (MLST), a protocol that was based on partial nucleotide sequences of housekeeping genes, is commonly used to differentiate between isolates of the same microbial species [19]. In recent decades MLST has been developed as a technique to examine the evolution and genetic population structure of bacteria [20–22]. It was initially evaluated for *Neisseria meningitidis* but has subsequently been extended to many bacterial species [20]. Most recently it has been used to characterize *Lactobacillus* species including *Lactobacillus casei* [23, 24], *Lactobacillus plantarum* [25], *Lactobacillus sanfranciscensis* [26], *Lactobacillus delbrueckii* and *Lactobacillus sakei* [27, 28]. However, until this study, MLST had not been applied to characterizing *L. fermentum* isolates.

Here we developed an MLST scheme based on 11 housekeeping gene fragments to characterize 203 isolates of *L. fermentum*. The aim of this study was to develop an effective MLST method for *L. fermentum* and use this to describe the diversity, genetic population structure and evolutionary origins of isolates within this species.

## Results

### Sequence diversity in *L. fermentum*

Partial sequences of 11 gene fragments (*clpX*, *dnaA*, *groEL*, *pyrG*, *rpoB*, *recA*, *murE*, *pepX*, *uvrC*, *dnaK* and *murC*) were determined (Table 1). The numbers of alleles, polymorphic sites, guanine-cytosine content, nucleotide diversity per site (*Pi*) and rate of *d*_*N*_*/d*_*S*_ value (*d*_*S*_ is the number of synonymous substitutions per synonymous site and *d*_*N*_ is the number of non-synonymous substitutions per non-synonymous site) were all determined (Table 2). Fragment sizes of the 11 housekeeping gene fragments, which ranged from 589 bp (*dnaA*) to 748 bp (*uvrC*), were used for MLST analysis. The number of alleles per gene fragment varied between seven (*groEL*) and 19 (*pepX*). Between six (*groEL*) and 22 (*pepX*) polymorphic sites were found for each gene fragment, and a total of 135 SNPs were identified. The mean guanine-cytosine content of the partial sequence of the 11 gene fragments varied between 48.26 % (*dnaA*) and 60.44 % (*pepX*). *Pi* was calculated for each individual gene fragment and varied from 0.00393 (*murC*) to 0.01421 (*dnaA*). The *d*_*N*_*/d*_*S*_ value for the 11 gene fragments varied between 0.0000 (*clpX, dnaA, dnaK,* and *recA*) and 0.2963 (*carB*).

**Table 1:** Genes and primers used for MLST

Gene	Position*	Primer	Sequence	Amplicon size (bp)
*clpX*	723485 - 724143	*clpX*/F	5’-CGCACGGAAGCAGAAAC-3’	659
		*clpX*/R	5’-GAGTCGGTCCCAAACCC-3’	
*dnaA*	410-998	*dnaA*/F	5’-ACCCGCTCCTGATTTACG-3’	589
		*dnaA*/R	5’-GCCTCGGTAGCCAGTTTG-3’	
*dnaK*	885345-886078	*dnaK*/F	5’-GACAACGGTCCGCTCCACT-3’	734
		*dnaK*/R	5’-TCGGCTTCTTCCTTCTTCTTCT-3’	
*groEL*	394357-395019	*groEL*/F	5’-CCGACAACGACAAGATGG-3’	663
		*groEL*/R	5’-CCAAGGCAGGGATAACG-3’	
*murC*	1501098-1500354	*murC*/F	5’-TTTGAAGCCGACGAATACC −3’	745
		*murC*/R	5’-CGATGTCCTCGCTACCC-3’	
*murE*	2086362-2085704	*murE*/F	5’-CTACCGCCAGCACTTCTT-3’	659
		*murE*/R	5’-GGTCCATCTGGGTGTTTAGC-3’	
*pepX*	1895783-1896506	*pepX*/F	5’-AAAGAAGACGAGCAACCAACC-3’	724
		*pepX*/R	5’-CGGAGTCCTTAGTCCCGATT-3’	
*pyrG*	246712-247420	*pyrG*/F	5’-TCATTGGGTCGGCTGTT-3’	709
		*pyrG*/R	5’-GGTCCATCCCTTGCTTTTG-3’	
*recA*	605296-605942	*recA*/F	5’-ATTGCCGACGCCCTGAT-3’	647
		*recA*/R	5’-TGCGGTTCGCCTTCCTT-3’	
*rpoB*	1719594-1718880	*rpoB*/F	5’-GAAGTTCCGCCGCTCTA-3’	715
		*rpoB*/R	5’-GGTCCCATCTGGCATGTAC-3’	
*uvrC*	727309-728056	*uvrC*/F	5’-TCGTCACCTCCTCCAATAA-3’	748
		*uvrC*/R	5’-TGGTTCGGTAATCCCTCC-3’	

*Positions correspond to complete genome sequence of *Lactobacillsu fermentum* IFO 3956

**Table 2:** Nucleotide and allelic diversity in 11 housekeeping gene fragments

Locus	Number of		Mean G + C content (mol%)	*Pi* ^1^	*d*_*N*_ */d*_*S*_^2^
	Alleles	Polymorphic sites			
*clpX*	11	11	53.24	0.00634	0
*dnaA*	9	12	48.26	0.01421	0
*dnaK*	12	13	56.5	0.0061	0
*groEL*	7	6	51.91	0.00416	0.2188
*murC*	12	12	55.16	0.00393	0.0812
*murE*	11	14	54.86	0.00768	0.0424
*pepX*	19	22	60.44	0.00769	0.2548
*pyrG*	10	10	54.61	0.0056	0.128
*recA*	12	11	56.27	0.00408	0
*rpoB*	11	7	52.63	0.00514	0.0683
*uvrC*	11	13	52.98	0.00618	0.1714

^1^ Mean pairwise nucleotide differences per site

^2^ *d*_*N*_ */d*_*S*_ = the ratio of nonsynonymous to synonymous substitutions

### Assignment of sequence types

An MLST protocol was developed for the 203 *L. fermentum* isolates based on 11 housekeeping gene fragments and used to identify STs (Additional file 1: Table S1). Fifty-seven STs were identified using combined data from the 11 gene fragments (ST-1 to ST-57) (Table S1). The 203 isolates were divided as follows: ST-4 (46 isolates); ST-5 (23 isolates); ST-23 (ten isolates); ST-37 (eight isolates); ST-6, 18 (six isolates each); ST-21, 26, 34, 55 (five isolates each); ST-12, 29, 38 (four isolates each); ST-1, 8, 11, 22, 24, 28, 31, 45, 48, 50, 56 (three isolates each); ST-9, 15, 25, 40, 43, 44 (two isolates each); the remaining 27 STs were each represented by a single isolate.

### Relatedness of *L. fermentum* isolates

**Fig. 1:**
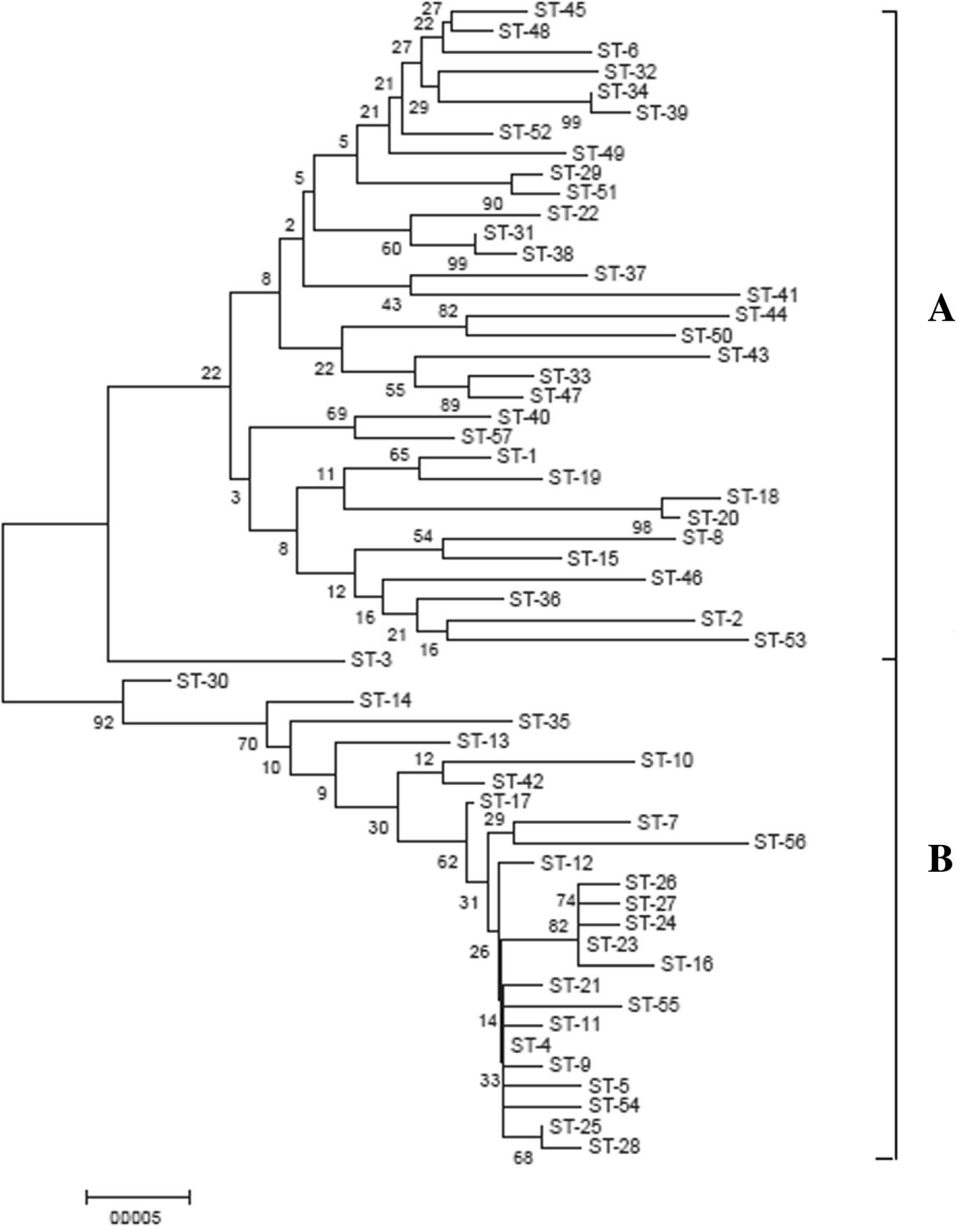
Neighbour-joining phylogenetic tree obtained from the concatenated nucleotide sequence of 57 STs. Bootstrap value are shown for all branches. The numbering in the figure refers to the ST. The two major phylogroups were designated as A and B

To infer the evolutionary relationships amongst *L. fermentum* isolates, a phylogenetic tree based on the MLST data was obtained using the Neighbour-joining (N-J) method (Fig. 1). In the phylogenetic tree built, all of the isolates investigated were well clustered into two major groups, A and B. Group A contained four clonal complexes (CCs, CC2-CC5) and 25 singletons, representing 78 *L. fermentem* isolates and one reference isolate (ST-57); most of the isolates (77 %) were isolated from acidic gruel. The reference isolate (ST-57), which was also found in Group A, was isolated from fermented plant material. Group B was the larger of the two groups and included CC1 and nine singletons; almost all the isolates (98 %) were isolated from different dairy products such as yoghurt, kurut, qula, fermented camels’ milk, koumiss, whey, etc.. The ancestral type for group B was ST-4, which contained 46 isolates originating from dairy products, in addition to IMAU70163, which was an isolate from acidic gruel.

### Recombination in *L. fermentum*

The values of *I*_A_ (the index of association) and *I*_*A*_^S^ (the standardised index of association) for the 11 gene fragments were 2.1424 and 0.2142 (*p* = 0.000), respectively. As the *I*_*A*_^S^ value was greater than 0, this indicates that the genes investigated in this study were close to linkage disequilibrium [29].

**Fig. 2:**
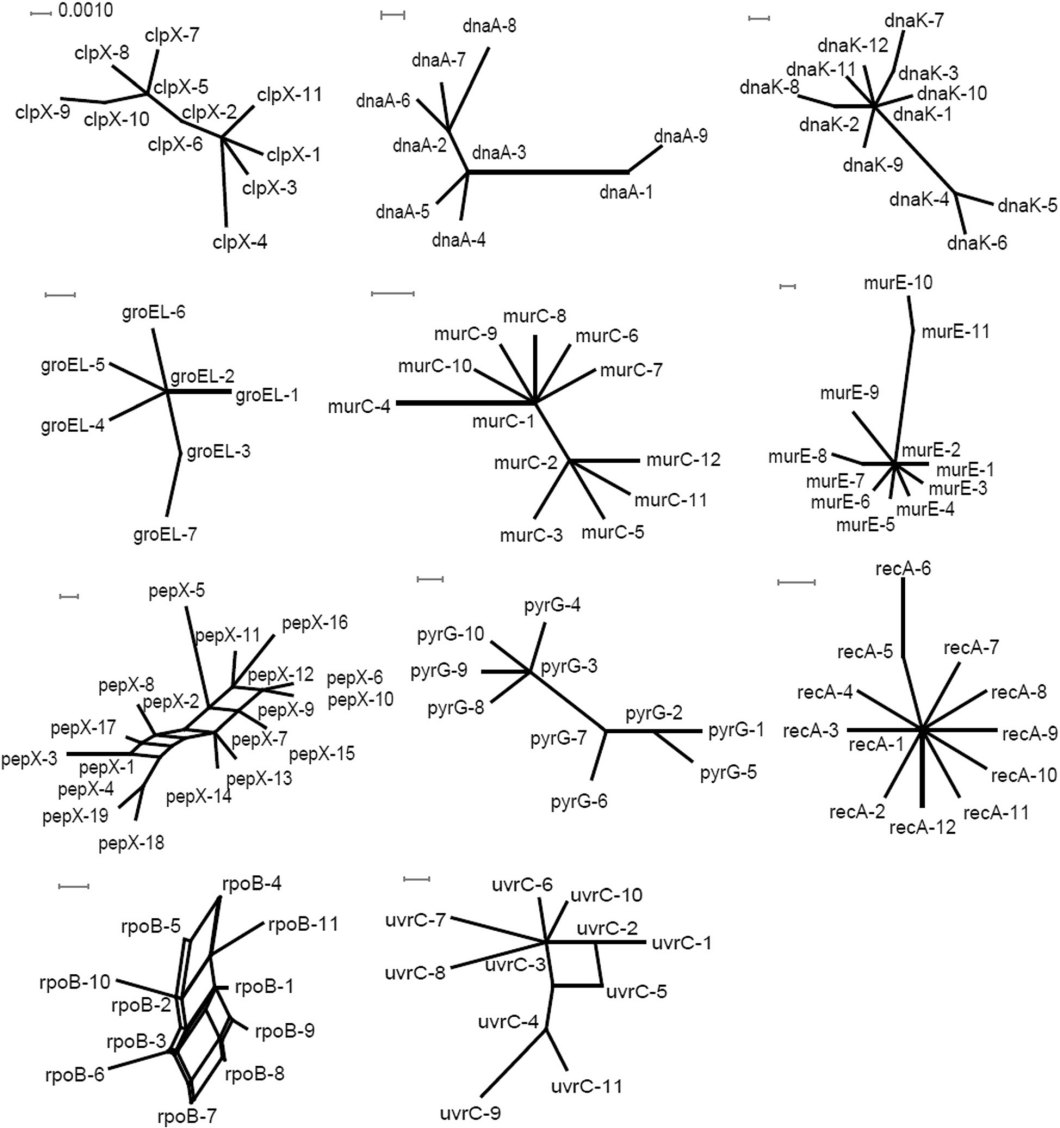
Split-decomposition analysis of 203 *L. fermentum* isolates with 11 housekeeping gene fragments. Multi-parallelogram formations indicate recombination events. Split-decomposition analysis of individual MLST loci. The numbering in the figure refers to allele numbers

Simultaneously, a split decomposition analysis examining evidence for recombination amongst the 203 *L. fermentum* isolates revealed different structures in the split graphs for all 11 gene fragments (Fig. 2). The split graphs for *pepX*, *uvrC*, and *rpoB* were network-like with parallelogram structures indicative of recombination in the evolutionary history of those genes. However, the split graphs for *groEL*, *dnaK, dnaA, murE, clpX, murC, recA* and *pyrG* were tree-like structures, indicative of a clonal descent for these genes and an absence of recombination.

**Fig. 3:**
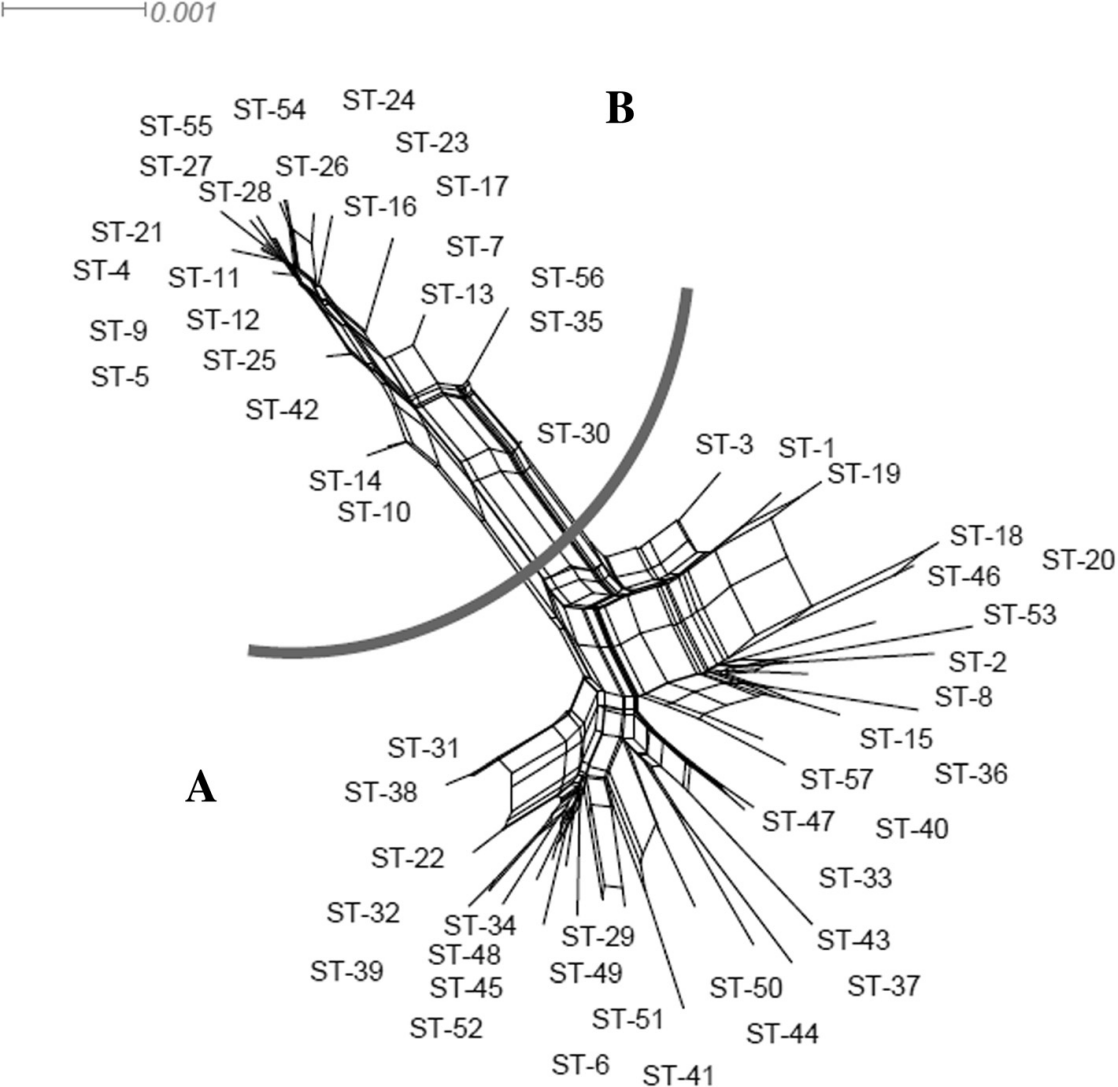
Split-decomposition analysis of 203 *L. fermentum* isolates with 11 housekeeping gene fragments. Multi-parallelogram formations indicate recombination events. Combined split-decomposition analysis of all 11 MLST loci. The numbering in the figure refers to the ST. Gray line was drawn as boundaries of each group

Based on split decomposition analysis the relationships amongst the 57 STs could be described as having a network-like structure with rays of different lengths (Fig. 3). The STs were divided into two main groups, A and B, and these groups were completely disconnected from each other. Parallelogram-shaped groupings were detected suggesting recombination events had occurred frequently. Isolates in group A were more distantly related to the ancestral isolate than the isolates in group B based on split decomposition analysis, suggesting that recombination had not occurred between isolates from the two groups, but that intergenic recombination may have occurred between isolates from the same group during their evolution. In addition, 57 STs indicated two distinct groups corresponding to group A and B in Fig. 1.

### Cluster analysis of the MLST data

**Fig. 4:**
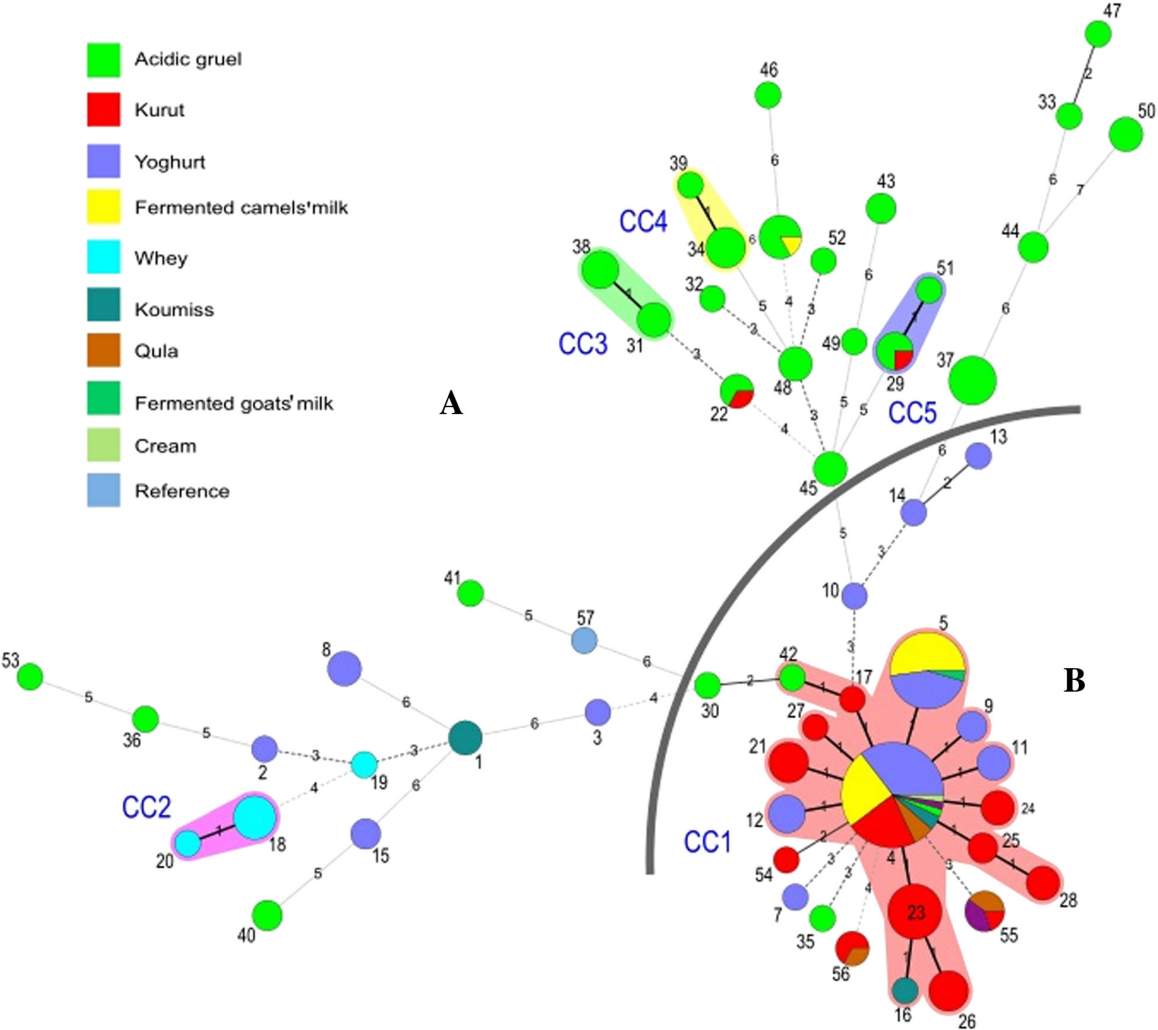
Minimum spanning tree analysis of 203 *L. fermentum* isolates based on allelic profiles of 11 gene fragments and according to food-type origin*.* Each circle represents the sequence type, the size of the circle is proportional to the number of isolates within any given ST. The strength of links are: black line = strong relationship; grey line = intermediate relationship; dotted line = weak relationship. STs belonging to the same clonal complex, are indicated by the surrounding shading. Gray line was drawn as boundaries of each group

Allelic profile-based phylogenetic analysis using the minimum spanning tree algorithm based on food-type and geographic origins, was used to explore genetic lineages amongst the *L. fermentum* isolates (Fig. 4). In this representation, isolates with the same allelic profile were assigned to the same circle, the size of the circle is proportional to the number of isolates with that unique profile. The clonal complexes (CCs) were confirmed as groups of STs sharing 9 of the 11 gene fragments. The 203 *L. fermentum* isolates evaluated were assigned to 57 STs that were distributed amongst five CCs and 34 unique STs (singletons).

Most of the isolates recovered from acidic gruel were absent from the largest CC, i.e. CC1. CC1 included 15 STs that mainly originated from traditional fermented dairy products from a wide range of geographic locations, including Mongolia, seven regions of Inner Mongolia, Tibet, Gansu, Sichuan, Yunnan, Qinghai and Xinjiang. The only exception was isolate IMAU70163, which belonged to CC1, but was isolated from acidic gruel. Within CC1, ST-4 had the largest number of isolates (46 = 23 % of the total) and was identified as the anscestral genotype surrounded by single-locus (ST-5, 9, 11, 12, 17, 21, 23, 24, 25 and 27), or two-locus variants (ST-16, 26, 28, and 42). All other CCs included only two STs, each with only a few isolates. CC2 included ST-18 and ST-20, and contained seven isolates from Yunnan. CC3 included ST-31 and ST-38, and contained seven isolates from Inner Mongolia. CC4 included ST-34 and ST-39, and contained six isolates from Inner Mongolia. CC5 included ST-29 and ST-51, and contained five isolates from Tibet and Inner Mongolia.

## Discussion

Multilocus sequence typing is considered to be the best method for typing isolates using the DNA sequences of selected housekeeping gene internal fragments [30]. In this study, we used the MLST method to analyze the natural diversity in *L. fermentum* based on the DNA sequences of 11 housekeeping gene fragments. The isolates evaluated here were isolated from a relatively large geographic area including Mongolia, four Chinese Provinces and two Autonomous regions of China, and from various naturally fermented dairy products and acidic gruel. These isolates provide the relevant information required for a better understanding of the population structure and phylogenetic relationships amongst 203 isolates of *L. fermentum*.

Nucleotide sequences-based methods for bacterial typing are the most unambiguous methods by which isolates of any microorganism can be identified [30, 31]. In this study, 203 *L. fermentum* isolates were divided into 57 STs, providing a clear indication of variability. We used the MLST method to compare nucleotide polymorphisms within regions of the 11 housekeeping gene fragments, which are under selective pressure to retain function. Housekeeping genes used in this study had a certain number of polymorphisms and hence were useful for designing the MLST protocol. A total of 131 polymorphic sites were detected in the 11 gene fragments giving a polymorphism rate of 1.72 % amongst the 7,592 nucleotides present in *L. fermentum* isolates. This value was higher than that seen for other LAB, such as *Oenococcus oeni*, which had 40 SNPs (0.99 %) amongst 4,040 base pairs sequenced [32]; and other microbes such as *Aspergillus fumigatus,* with 41 SNPs (1.35 %) amongst 3,038 base pairs sequenced [33]. This result also indicated that, because most of the gene fragments used in this study had high nucleotide genetic diversity, they also had a strong discriminatory ability.

The value of *Pi* varied from 0.00393 (*murC*) to 0.01421 (*dnaA*) for each gene fragment. Except for *dnaA*, most fragments showed similar nucleotide diversity of between 0.00393 and 0.00769. Our estimate of *Pi* was similar to those obtained for *L. plantarum*, which ranged from 0.0004 to 0.0072 [25] and *L. delbrueckii*, which ranged from 0.0051 to 0.0096 [27]. These comparisons demonstrate the relatively high nucleotide diversity in these species and in the *L. fermentum* isolates used in this study. This relatively high nucleotide diversity in *L. fermentum* is possibly as a result of recombination by natural transformation and a low frequency of mutation. In our genetic analysis, the *d*_*N*_*/d*_*S*_ ratios of the 11 gene fragments were all less than 1, suggesting purifying selection as expected from relatively conserved housekeeping genes. In particular the values for *clpX*, *dnaA*, *dnaK* and *recA* were zero, indicating that the amino acid composition of these genes did not change. Similar results have also been found in other studies, supporting our conclusion that these housekeeping genes are all under stabilizing selection [34, 35].

The phylogenetic tree constructed from the concatenated sequences of the 11 gene fragments indicated that the 203 *L. fermentum* isolates formed two distinct groups (A and B); partitioning of the two groups was as a result of evolutionary changes in the *clpX*, *dnaA*, *dnaK*, *groEL*, *murC, murE*, *pepX*, *pyrG*, *recA*, *rpoB* and *uvrC* gene sequences, as was clearly visible in the N-J phylogenetic tree constructed (Fig. 1). This showed a strong evolutionary tendency for *L. fermentum* isolates from the same geographic areas, including Mongolia, a number of Chinese Provinces and an Autonomous region, and from the same naturally fermented products, to be similar. MLST analyses were valuable in identifying these differences in variation within the *L. fermentum* genome. Hence, *L. fermentum* isolates that were included in the same group shared similarities in genome organization. Notably, about eight of ten isolates from acidic gruel were grouped together within group A and almost all the isolates from fermented dairy products were grouped together within group B. The minimum spanning tree analysis supported the existence of two groups of isolates (Fig. 4). Although MLST analysis of 203 *L. fermentum* isolates indicated two groups here, we cannot exclude the possible that more complex population structures might be identified if a larger number of isolates from more diverse sources was used. In future work, MLST will be a useful tool to examine possible relationships amongst *L. fermentum* isolates, while simultaneously aiding the selection of industrial isolates with greatest potential for the production of fermented food.

The analysis of population structure of *L. fermentum* isolates indicated substantial recombination phenomena. The *I*_*A*_^S^ value for the 11 gene fragments from 203 *L. fermentum* isolates was calculated as 0.2142 (*p* = 0.000), which is indicative of a weak clonal population structure (i.e. linkage disequilibrium). Several studies have already shown that LAB isolated from fermented milk are structured in rather clonal populations, for example, Xu *et al*., (2013) investigated 12 housekeeping genes in *Lactococcus lactis*; the *I*_*A*_^S^ value was 0.3038 and indicative of a clonal population [35].

Split-decomposition analysis also provided evidence of intraspecies recombination that could play a role in generating genotypic diversity amongst isolates according to the allelic profiles of the isolates evaluated. Split graphs for individual loci indicated tree-like or network-like structures, suggesting that some genes were affected by intraspecies recombination (Fig. 2). Simultaneously, a split graph representation of the concatenated sequence of the 11 loci clearly indicated that two groups and their descents orginated from intraspecies recombination (Fig. 3). The concatenated dendogram corresponded well with the allele-based dendogram (Fig. 1). However, some small differences were found between the concatenated dendogram and the allele-based dendogram. For example, ST-25 was positioned further away from ST-28 in the latter than the former. Although intraspecies recombination occurs frequently, isolates from fermented dairy products in group B and isolates from acidic gruel in group A in both the N-J phylogenetic tree (Fig. 1) and the combined split graph (Fig. 3), seem to be clonal. These results suggest that isolates from fermented dairy products and acidic gruel may have a common recent ancestor. In addition, the minimum spanning tree analysis result confirmed the above assumption that these isolates have a common recent ancestor (Fig. 4).

The clustering of isolates by food-type origin was evident in the minimum spanning tree (Fig. 4) demonstrating strong patterns of specificity for source within the 203 *L. fermentum* isolates evaluated. The diversity found amongst isolates from fermented dairy products differed from that found amongst isolates from acidic gruel. Almost all of the isolates from fermented dairy products were assigned to the largest CC, CC1, whereas isolates from acidic gruel were dispersed among a larger number of STs. Although the difference between isolates from fermented dairy products and acidic gruel was significant, we considered that evolution of *L. fermentum* was not correlated with food-type origins. We suggest that a more simple explanation is that the ecological niches from which the isolates were sampled are very narrow (several types of dairy products and acidic gruels). As *L. fermentum* is a ubiquitous bacterium found in many different types of food and in animal faeces, it is likely that isolates from dairy products are only a small proportion of the natural diversity within this species, particularly as they would be selected for particular attributes associated with the fermentation of food. We also found no association between STs and geographical origin (data not shown).

MLST data are usually subdivided into nonoverlapping groups of related STs or CCs using an eBURST algorithm approach to determine the most parsimonious patterns of descent of isolates within each CC from the predicted founder [36]. Here we also used the eBURST algorithm to analyze the MLST data, and found that ST-4 inhabited a central location, and that other STs had relatives that were derived from the ubiquitous STs themselves and exhibited a limited genetic diversity, as has been found in other studies [37, 38]. The great majority of STs in CC1 are single-locus variants (SLVs) of ST-4, which strongly supported primary founders. Furthermore, CC1 to CC5 were clearly formed based on food-type origins. For example, three of the seven CCs (CC1 to CC2) consisted exclusively of isolates from fermented dairy products, with the exception of only one isolate (IMAU70056), whereas three of five CCs (CC3 to CC5) consisted exclusively of isolates from acidic gruel, with the exception of only one isolate (IMAU60167). Furthermore, we found that the *L. fermentum* isolates in CC1, which were almost exclusively isolated from fermented dairy products, were located centrally, with isolates from acidic gruel being distributed around CC1 and not centrally (Additional file 2: Figure S1).

The genus *Lactobacillus*, together with the genera *Paralactobacillus* and *Pediococcus*, is the largest group in the *Lactobacillaceae* and order *Lactobacillales* in the *Firmicutes* [39]. The species *L. fermentum* and *Lactobacillus reuteri* are phylogenetically closely related and are regarded together as the *L. reuteri* group. Since Maiden *et al.,* (1998) first described an MLST technique for *N. meningitides,* MLST methods have been used to differentiate between isolates within species from the genus *Lactobacillus* including *L. casei*, *L. plantarum*, *L. sanfranciscensis*, *L. delbrueckii*, and *L. sakei*, [23–28]. To date, the MLST schemes used in these studies were distinct from each other because the isolates used in each study came from different habitats, and different housekeeping genes were selected for analysis. In these studies, 41 isolates of *L. delbrueckii* were assigned to 34 STs [27]; 52 isolates of *L. casei* (*L. paracasei*) were assigned to 31 STs [24]; 232 isolates of *L. sakei* were assigned to 116 unique STs of which all isolates had evolved into three clades, each with a unique population structure [28]. In our study, we used MLST to identify 57 STs within a *L. fermentum* population of 203 isolates. Our research indicated that this species had a clonal population structure and a pattern of diversity different from the previously mentioned species. Moreover, two groups, each with a unique population structure, were identified amongst *L. fermentum* isolates (including the reference isolate *L. fermentum* IFO 3956). The *L. fermentum* MLST schemes in this study demonstrate that MLST is a useful tool for discrimination between isolates, and furthermore provides a method to analyze the evolution and population structure of *Lactobacillius* species from various sources.

## Conclusions

In this study, a novel MLST protocol was used to investigate the population genetic structure and evolutionary characteristics of *L. fermentum*. The MLST protocol presented provided high discriminatory power for molecular typing of *L. fermentum* isolates. Furthermore, we were able to shed light on how this species has evolved into two unique groups. Using a large number of isolates allowed us to better interpret the possible ecological differences underlying the two branches observed. Finally, we also found that the evolution of *L. fermentum* STs was not correlated with geography or food-type origin. Taken together, our results indicate that MLST of *L. fermentum* was an easy and valuable tool that, together with the construction of an MLST database, will contribute to further detailed studies on the evolution and population genetics of *L. fermentum*.

## Methods

### Bacterial isolates and growth conditions

A total of 203 *L. fermentum* isolates, from the Lactic Acid Bacteria Collection Center of the Inner Mongolia Agricultural University (LABCC), were used in this study (Table S1). These isolates came from various sources including yoghurt, kurut, koumiss, fermented camels’ milk, fermented goats’ milk, qula, whey, cream, acidic gruel and other traditional foods from Mongolia; the Provinces of Sichuan, Qinghai, Yunnan and Gansu from the P.R of China; and the Autonomous Regions of Inner Mongolia, Xinjiang and Tibet from the P.R of China. All isolates were cultured under anaerobic conditions in de Man Rogosa Sharpe broth (MRS) (Becton, Dickinson Co., Sparks, Md., USA) at 37 **°**C for 24 h. The reference isolate, *L. fermentum* IFO 3956 which was originally obtained from fermented plant material, was obtained from the NCBI genome database (http://www.ncbi.nlm.nih.gov/genome/).

### DNA extraction

Bacterial DNA was extracted from all isolates of *L. fermentum* as described previously [40]. Briefly, the bacterial cells were precipitated out of suspension by centrifugation (8,000 × *g*, 3 min, 4 **°**C) after overnight incubation in MRS broth at 37 **°**C. The pellet was subjected to freeze-thaw cycles to ensure cell lysis. After thoroughly washing the pellet with phosphate-buffered saline (PBS), 10 % SDS and proteinase-K solution (20 mg/ml) were added and incubated in a shaking incubator at 200 rpm and 37 **°**C overnight. Subsequently, 0.7 M NaCl and 10 % cetyl trimethyl ammonium bromide (CTAB) were added and incubated at 65 **°**C for a further 20 minutes. Protein contaminants were removed by phenol extraction and the DNA was precipitated with an equal volume of ice-cold isopropanol, and then washed thoroughly in 70 % (v/v) ice-cold ethanol. The final DNA concentration was determined by recording its optical density at 260 and 280 nm, respectively, using a NanoDrop ND-1000 spectrophotometer (NanoDrop Technologies, Wilmington, DE, USA).

### Selection of housekeeping genes for MLST

General criteria for selection of housekeeping genes include their location on the chromosome, the function of the encoded proteins, their presence in all isolates as a single copy and that gene size is at least 1 kb [23]. Based on these criteria and by examining the gene sequences of *L. fermentum* IFO 3956, 11 housekeeping genes (*clpX*, *dnaA*, *dnaK*, *groEL*, *murC, murE*, *pepX*, *pyrG*, *recA*, *rpoB* and *uvrC*) were selected. Among them, ten housekeeping genes (*clpX*, *dnaA*, *dnaK*, *groEL*, *murC, murE*, *pepX*, *pyrG*, *recA* and *uvrC*) were selected based on the results of a previous study on *Lactobacillus helveticus* [41], the remaining locus (*rpo*B) was based on *Lactobacillus sakei* [28].

These housekeeping genes were also described from the variable regions in the *L. fermentum* IFO 3956 genome sequence [42]: *clpX* encoding ATP-dependent protease ATP-binding subunit ClpX (YP_001843436), *dnaA* encoding chromosomal replication initiation protein (YP_001842817.1), *groEL* encoding chaperonin GroEL (YP_001843142.1), *pyrG* encoding CTP synthetase (YP_001843018.1), *rpoB* encoding DNA-directed RNA polymerase subunit beta (YP_001844339.1), *recA* encoding recombinase A (YP_001843327.1), *murE* encoding UDP-N-acetylmuramoylalanyl-D-glutamate-2, 6-diaminopimelate ligase (YP_001844650.1), *pepX* encoding x-prolyl-dipeptidyl aminopeptidase (YP_001844484.1), *uvrC* encoding excinuclease ABC subunit C (YP_001843441.1), *dnaK* encoding molecular chaperone DnaK (YP_001843567.1) and *murC* encoding UDP-N-acetylmuramate-L-alanine ligase (YP_001844136.1).

### PCR amplification and nucleotide sequencing

In this study, the MLST protocol was modified to study the population structure of *L. fermentum* using the 11 housekeeping genes selected*.* Bacterial DNA from the 203 isolates of *L. fermentum* was used as the template for amplification of the 11 housekeeping genes. Primers were designed based on the internal fragments of the 11 gene fragments with Primer Premier 6.0 software (Table 1). Primers targeting the internal fragments of each gene ranged from 589 to 748 bp in size. The PCR mixture (10 μl) contained 0.08 μl Taq polymerase (5 U/μl, Takara, Tokyo), 1 μl 10 × PCR buffer (Mg^2+^ free), 0.8 μl dNTPs (2.5 mM each), 0.8 μl MgCl_2_ (25 mM), 0.4 μl forward primer (10 μM), 0.4 μl reverse primer (10 μM), 1 μl genomic DNA (10–50 ng/μl) and 5.52 μl dH_2_O. For *clpX*, *dnaA*, *groEL*, *pyrG*, *rpoB*, *recA*, *murE*, *pepX uvrC*, *dnaK* and *murC,* the PCR procedure was done under the following conditions: 94 **°**C for 5 sec, 30 cycles of amplification which included 95 **°**C for 60 sec, 50 **°**C for 45 sec, 72 **°**C for 60 sec and then annealing at 72 **°**C for 10 min. Sequencing of the PCR products was done by the Shanghai Sangni Biosciences Corporation (Shanghai, China). Every isolate was sequenced by Sanger dideoxy sequencing, using both DNA strands. Each MLST allele fragment was sequenced on both nucleotide strands. After obtaining sequences for each isolate, we trimmed, aligned and adjusted these sequences using the MEGA 5.0 software package, and these sequences had forward and reverse sequences, which guaranteed their validity.

### Descriptive analysis of MLST sequence data

The sequence data obtained for the 11 housekeeping gene fragments were imported into BioNumerics software (version 6.0, Applied-Maths, Sint Maartens-Latem, Belgium) and allele numbers per gene fragment were obtained. Afterwards, the combination of 11 allele numbers per isolate was assigned to an allelic profile, i.e. a sequence type [43]. Isolates with the same ST had identical allelic profiles. To analyze the micro-evolutionary processes linking STs from all isolates, a minimum-spanning tree was constructed with Prims's algorithm in the BioNumerics software according to region and source separation (version 6.0, Applied-Maths, Sint Maartens-Latem, Belgium).

The guanine-cytosine content, *d*_*N*_*/d*_*S*_ ratio, nucleotide diversity, the number of polymorphic sites and single nucleotide polymorphisms (SNPs) in the 11 housekeeping gene fragments for each isolate, were calculated using DnaSP 5.0 [44, 45] and START 2.0 (http://www.mlst.net/links/software.asp) [46].

Phylogenetic trees were constructed using the neighbour-joining method in MEGA version 5.0 software (version 5.0, www.megasoftware.net). Identifying the relationships between individual STs and their clustering into clonal complexes was achieved using eBURST (Based Upon Related Sequence Types) V 3.0 software (http://eburst.mlst.net) in relation to their number of SLVs, double-locus variants (DLVs) or triple-locus variants (TLVs) [30, 37]. Split decomposition analysis was done with SplitsTree 4.0 and START 2.0 software on the MLST website (http://eburst.mlst.net/) [37]. The level of linkage disequilibrium between the 11 alleles of 203 isolates was calculated in START 2.0 software by determining the value of *I*_*A*_^S^ [47].

### Nucleotide sequence accession numbers

The partial sequences of the 11 MLST loci used in this study have been deposited in the GenBank/EMBL databases under accession numbers KR078446-KR080061 and KP224504-KP225109.

## Additional files

Additional file 1: Table S1. Allelic profiles of 203 *Lactobacillus fermentum* isolates. The information on isolates used in this study are listed and identified. Additional file 2: Figure S1. eBURST analysis of 203 *L. fermentum* isolates used in this study. In the eBURST diagram, five clonal and thirty-four singletons are shown. The primary founders of eBURST groups are positioned centrally in the cluster and labeled in blue, and the subgroup founders are shown in yellow. Dots indicate sequence type and lines connect single-locus variants, which are STs that differ in only one of the 11 housekeeping gene fragments. Boxed numbers indicate STs found in acidic gruel, the other numbers indicated STs found in dairy products.

## Abbreviations

CCs: Clonal Complexes
*d*_*N*_: Non-synonymous Substitutions
*d*_*S*_: Synonymous Substitutions
FISH: Fluorescence in Situ Hybridization
*I*_*A*_^S^: Standardized Index of Association
LAB: Lactic Acid Bacterium
MLST: Multilocus Sequence Typing
PFGE-RFLP: Pulsed-field Gel Electrophoresis with Restriction Fragment Length Polymorphism
RAPD-PCR: Randomly Amplified Polymorphic DNA
SNP: single nucleotide polymorphism
ST: Sequence Type.
