# Erratum: The evolution and population structure of Lactobacillus fermentum from different naturally fermented products as determined by multilocus sequence typing (MLST)

**DOI:** 10.1186/s12866-016-0651-5

**Published:** 2016-03-22

**Authors:** Tong Dan, Wenjun Liu, Yuqin Song, Haiyan Xu, Bilige Menghe, Heping Zhang, Zhihong Sun

**Affiliations:** Key Laboratory of Dairy Biotechnology and Engineering, Education Ministry of P. R. China, Department of Food Science and Engineering, Inner Mongolia Agricultural University, Hohhot, 010018 P. R. China

## Erratum

The original version of this article [1] unfortunately contained a mistake in the section entitled “Nucleotide sequence accession numbers”. The accession numbers of two housekeeping genes (clpX and pyrG)used in this study were wrongly provided. KR078446-KR078647 should read KU512980 - KU513181 and KR079456 - KR079657 should read KU513182 - KU513383.

The correct text is provided below:

## Nucleotide sequence accession numbers

The partial sequences of the 11 MLST loci used in this study have been deposited in the GenBank/EMBL databases under accession numbers KR078648-KR079455,KR079658-KR080061,KP224504-KP225109, KU512980 - KU513181 and KU513182 - KU513383.
