# Supplementary material for: The evolution and population structure of Lactobacillus fermentum from different naturally fermented products as determined by multilocus sequence typing (MLST)
Source: BMC Microbiol. 2015 May 20;15:107. doi: 10.1186/s12866-015-0447-z (PMC4437502; doi:10.1186/s12866-015-0447-z)
Supplement: Additional file 1: Table S1. — Allelic profiles of 203 Lactobacillus fermentum isolates. The information on isolates used in this study are listed and identified. [file 12866_2015_447_MOESM1_ESM.docx]

| **Isolate no.** | **Sequence Type** | **Clonal Complex** | **Allelic profile** | | | | | | | | | | | **Isolation source** | **Collection place** | **Year of isolation** |
| --- | --- | --- | --- | --- | --- | --- | --- | --- | --- | --- | --- | --- | --- | --- | --- | --- |
|  |  |  | ***pyr*G** | ***rpo*B** | ***gro*EL** | ***rec*A** | ***clp*X** | ***mur*E** | ***pep*X** | ***uvr*C** | ***dna*K** | ***mur*C** | ***dna*A** |  |  |  |
| IMAU10687 | ST1 | singleton | 4 | 1 | 2 | 10 | 2 | 11 | 6 | 4 | 1 | 6 | 2  2  2  2  2  2  2  2  2  2  2  2  2  2  2  2  2  2  2  2  2  2  2  2  3  3  3  3  3  2  2  2  2  2  2  2  2  2  2  2  2  2  4  4  4  2  2  3  2  2  5  5  5  5  2  2  4  4  4  4  2  3  4  4  4  4  4  4  4  3  2  2  2  2  2  2  2  2  2  2  2  2  3  3  3  2  2  1  2  2  2  2  2  2  2  2  2  2  2  2  2  2  2  2  2  2  2  2  2  2  2  3  3  3  3  3  3  3  3  3  6  3  3  3  3  3  4  4  4  4  4  4  4  4  4  4  3  3  3  3  3  2  2  3  3  7  7  6  6  3  3  3  9  6  3  3  3  10  8  8  8  3  3  3  9  2  2  2  2  2  2  2  1  1  1 | Koumiss | Inner Mongolia | 2009 |
| IMAU10692 | ST1 | singleton | 4 | 1 | 2 | 10 | 2 | 11 | 6 | 4 | 1 | 6 | 2  2  2  2  2  2  2  2  2  2  2  2  2  2  2  2  2  2  2  2  2  2  2  2  3  3  3  3  3  2  2  2  2  2  2  2  2  2  2  2  2  2  4  4  4  2  2  3  2  2  5  5  5  5  2  2  4  4  4  4  2  3  4  4  4  4  4  4  4  3  2  2  2  2  2  2  2  2  2  2  2  2  3  3  3  2  2  1  2  2  2  2  2  2  2  2  2  2  2  2  2  2  2  2  2  2  2  2  2  2  2  3  3  3  3  3  3  3  3  3  6  3  3  3  3  3  4  4  4  4  4  4  4  4  4  4  3  3  3  3  3  2  2  3  3  7  7  6  6  3  3  3  9  6  3  3  3  10  8  8  8  3  3  3  9  2  2  2  2  2  2  2  1  1  1 | Koumiss | Inner Mongolia | 2009 |
| IMAU10705 | ST1 | singleton | 4 | 1 | 2 | 10 | 2 | 11 | 6 | 4 | 1 | 6 | 2  2  2  2  2  2  2  2  2  2  2  2  2  2  2  2  2  2  2  2  2  2  2  2  3  3  3  3  3  2  2  2  2  2  2  2  2  2  2  2  2  2  4  4  4  2  2  3  2  2  5  5  5  5  2  2  4  4  4  4  2  3  4  4  4  4  4  4  4  3  2  2  2  2  2  2  2  2  2  2  2  2  3  3  3  2  2  1  2  2  2  2  2  2  2  2  2  2  2  2  2  2  2  2  2  2  2  2  2  2  2  3  3  3  3  3  3  3  3  3  6  3  3  3  3  3  4  4  4  4  4  4  4  4  4  4  3  3  3  3  3  2  2  3  3  7  7  6  6  3  3  3  9  6  3  3  3  10  8  8  8  3  3  3  9  2  2  2  2  2  2  2  1  1  1 | Koumiss | Inner Mongolia | 2009 |
| IMAU10712 | ST2 | singleton | 4 | 7 | 2 | 6 | 3 | 3 | 6 | 4 | 1 | 6 | 2  2  2  2  2  2  2  2  2  2  2  2  2  2  2  2  2  2  2  2  2  2  2  2  3  3  3  3  3  2  2  2  2  2  2  2  2  2  2  2  2  2  4  4  4  2  2  3  2  2  5  5  5  5  2  2  4  4  4  4  2  3  4  4  4  4  4  4  4  3  2  2  2  2  2  2  2  2  2  2  2  2  3  3  3  2  2  1  2  2  2  2  2  2  2  2  2  2  2  2  2  2  2  2  2  2  2  2  2  2  2  3  3  3  3  3  3  3  3  3  6  3  3  3  3  3  4  4  4  4  4  4  4  4  4  4  3  3  3  3  3  2  2  3  3  7  7  6  6  3  3  3  9  6  3  3  3  10  8  8  8  3  3  3  9  2  2  2  2  2  2  2  1  1  1 | Yoghurt | Inner Mongolia | 2009 |
| IMAU10716 | ST3 | singleton | 4 | 2 | 2 | 1 | 2 | 11 | 12 | 11 | 10 | 1 | 2  2  2  2  2  2  2  2  2  2  2  2  2  2  2  2  2  2  2  2  2  2  2  2  3  3  3  3  3  2  2  2  2  2  2  2  2  2  2  2  2  2  4  4  4  2  2  3  2  2  5  5  5  5  2  2  4  4  4  4  2  3  4  4  4  4  4  4  4  3  2  2  2  2  2  2  2  2  2  2  2  2  3  3  3  2  2  1  2  2  2  2  2  2  2  2  2  2  2  2  2  2  2  2  2  2  2  2  2  2  2  3  3  3  3  3  3  3  3  3  6  3  3  3  3  3  4  4  4  4  4  4  4  4  4  4  3  3  3  3  3  2  2  3  3  7  7  6  6  3  3  3  9  6  3  3  3  10  8  8  8  3  3  3  9  2  2  2  2  2  2  2  1  1  1 | Yoghurt | Inner Mongolia | 2009 |
| IMAU20031 | ST4 | CC1 | 2 | 2 | 2 | 1 | 4 | 11 | 1 | 2 | 4 | 1 | 1  2  2  2  2  2  2  2  2  2  2  2  2  2  2  2  2  2  2  2  2  2  2  2  3  3  3  3  3  2  2  2  2  2  2  2  2  2  2  2  2  2  4  4  4  2  2  3  2  2  5  5  5  5  2  2  4  4  4  4  2  3  4  4  4  4  4  4  4  3  2  2  2  2  2  2  2  2  2  2  2  2  3  3  3  2  2  1  2  2  2  2  2  2  2  2  2  2  2  2  2  2  2  2  2  2  2  2  2  2  2  3  3  3  3  3  3  3  3  3  6  3  3  3  3  3  4  4  4  4  4  4  4  4  4  4  3  3  3  3  3  2  2  3  3  7  7  6  6  3  3  3  9  6  3  3  3  10  8  8  8  3  3  3  9  2  2  2  2  2  2  2  1  1  1 | Fermented camels’ milk | Mongolia | 2006 |
| IMAU20041 | ST4 | CC1 | 2 | 2 | 2 | 1 | 4 | 11 | 1 | 2 | 4 | 1 | 1  2  2  2  2  2  2  2  2  2  2  2  2  2  2  2  2  2  2  2  2  2  2  2  3  3  3  3  3  2  2  2  2  2  2  2  2  2  2  2  2  2  4  4  4  2  2  3  2  2  5  5  5  5  2  2  4  4  4  4  2  3  4  4  4  4  4  4  4  3  2  2  2  2  2  2  2  2  2  2  2  2  3  3  3  2  2  1  2  2  2  2  2  2  2  2  2  2  2  2  2  2  2  2  2  2  2  2  2  2  2  3  3  3  3  3  3  3  3  3  6  3  3  3  3  3  4  4  4  4  4  4  4  4  4  4  3  3  3  3  3  2  2  3  3  7  7  6  6  3  3  3  9  6  3  3  3  10  8  8  8  3  3  3  9  2  2  2  2  2  2  2  1  1  1 | Fermented camels’ milk | Mongolia | 2006 |
| IMAU20044 | ST4 | CC1 | 2 | 2 | 2 | 1 | 4 | 11 | 1 | 2 | 4 | 1 | 1  2  2  2  2  2  2  2  2  2  2  2  2  2  2  2  2  2  2  2  2  2  2  2  3  3  3  3  3  2  2  2  2  2  2  2  2  2  2  2  2  2  4  4  4  2  2  3  2  2  5  5  5  5  2  2  4  4  4  4  2  3  4  4  4  4  4  4  4  3  2  2  2  2  2  2  2  2  2  2  2  2  3  3  3  2  2  1  2  2  2  2  2  2  2  2  2  2  2  2  2  2  2  2  2  2  2  2  2  2  2  3  3  3  3  3  3  3  3  3  6  3  3  3  3  3  4  4  4  4  4  4  4  4  4  4  3  3  3  3  3  2  2  3  3  7  7  6  6  3  3  3  9  6  3  3  3  10  8  8  8  3  3  3  9  2  2  2  2  2  2  2  1  1  1 | Fermented camels’ milk | Mongolia | 2006 |
| IMAU20080 | ST4 | CC1 | 2 | 2 | 2 | 1 | 4 | 11 | 1 | 2 | 4 | 1 | 1  2  2  2  2  2  2  2  2  2  2  2  2  2  2  2  2  2  2  2  2  2  2  2  3  3  3  3  3  2  2  2  2  2  2  2  2  2  2  2  2  2  4  4  4  2  2  3  2  2  5  5  5  5  2  2  4  4  4  4  2  3  4  4  4  4  4  4  4  3  2  2  2  2  2  2  2  2  2  2  2  2  3  3  3  2  2  1  2  2  2  2  2  2  2  2  2  2  2  2  2  2  2  2  2  2  2  2  2  2  2  3  3  3  3  3  3  3  3  3  6  3  3  3  3  3  4  4  4  4  4  4  4  4  4  4  3  3  3  3  3  2  2  3  3  7  7  6  6  3  3  3  9  6  3  3  3  10  8  8  8  3  3  3  9  2  2  2  2  2  2  2  1  1  1 | Fermented camels’ milk | Mongolia | 2006 |
| IMAU20651 | ST4 | CC1 | 2 | 2 | 2 | 1 | 4 | 11 | 1 | 2 | 4 | 1 | 1  2  2  2  2  2  2  2  2  2  2  2  2  2  2  2  2  2  2  2  2  2  2  2  3  3  3  3  3  2  2  2  2  2  2  2  2  2  2  2  2  2  4  4  4  2  2  3  2  2  5  5  5  5  2  2  4  4  4  4  2  3  4  4  4  4  4  4  4  3  2  2  2  2  2  2  2  2  2  2  2  2  3  3  3  2  2  1  2  2  2  2  2  2  2  2  2  2  2  2  2  2  2  2  2  2  2  2  2  2  2  3  3  3  3  3  3  3  3  3  6  3  3  3  3  3  4  4  4  4  4  4  4  4  4  4  3  3  3  3  3  2  2  3  3  7  7  6  6  3  3  3  9  6  3  3  3  10  8  8  8  3  3  3  9  2  2  2  2  2  2  2  1  1  1 | Yoghurt | Mongolia | 2009 |
| IMAU20652 | ST4 | CC1 | 2 | 2 | 2 | 1 | 4 | 11 | 1 | 2 | 4 | 1 | 1  2  2  2  2  2  2  2  2  2  2  2  2  2  2  2  2  2  2  2  2  2  2  2  3  3  3  3  3  2  2  2  2  2  2  2  2  2  2  2  2  2  4  4  4  2  2  3  2  2  5  5  5  5  2  2  4  4  4  4  2  3  4  4  4  4  4  4  4  3  2  2  2  2  2  2  2  2  2  2  2  2  3  3  3  2  2  1  2  2  2  2  2  2  2  2  2  2  2  2  2  2  2  2  2  2  2  2  2  2  2  3  3  3  3  3  3  3  3  3  6  3  3  3  3  3  4  4  4  4  4  4  4  4  4  4  3  3  3  3  3  2  2  3  3  7  7  6  6  3  3  3  9  6  3  3  3  10  8  8  8  3  3  3  9  2  2  2  2  2  2  2  1  1  1 | Yoghurt | Mongolia | 2009 |
| IMAU60104 | ST4 | CC1 | 2 | 2 | 2 | 1 | 4 | 11 | 1 | 2 | 4 | 1 | 1  2  2  2  2  2  2  2  2  2  2  2  2  2  2  2  2  2  2  2  2  2  2  2  3  3  3  3  3  2  2  2  2  2  2  2  2  2  2  2  2  2  4  4  4  2  2  3  2  2  5  5  5  5  2  2  4  4  4  4  2  3  4  4  4  4  4  4  4  3  2  2  2  2  2  2  2  2  2  2  2  2  3  3  3  2  2  1  2  2  2  2  2  2  2  2  2  2  2  2  2  2  2  2  2  2  2  2  2  2  2  3  3  3  3  3  3  3  3  3  6  3  3  3  3  3  4  4  4  4  4  4  4  4  4  4  3  3  3  3  3  2  2  3  3  7  7  6  6  3  3  3  9  6  3  3  3  10  8  8  8  3  3  3  9  2  2  2  2  2  2  2  1  1  1 | Kurut | Tibet | 2007 |
| IMAU20043 | ST4 | CC1 | 2 | 2 | 2 | 1 | 4 | 11 | 1 | 2 | 4 | 1 | 1  2  2  2  2  2  2  2  2  2  2  2  2  2  2  2  2  2  2  2  2  2  2  2  3  3  3  3  3  2  2  2  2  2  2  2  2  2  2  2  2  2  4  4  4  2  2  3  2  2  5  5  5  5  2  2  4  4  4  4  2  3  4  4  4  4  4  4  4  3  2  2  2  2  2  2  2  2  2  2  2  2  3  3  3  2  2  1  2  2  2  2  2  2  2  2  2  2  2  2  2  2  2  2  2  2  2  2  2  2  2  3  3  3  3  3  3  3  3  3  6  3  3  3  3  3  4  4  4  4  4  4  4  4  4  4  3  3  3  3  3  2  2  3  3  7  7  6  6  3  3  3  9  6  3  3  3  10  8  8  8  3  3  3  9  2  2  2  2  2  2  2  1  1  1 | Fermented camels’ milk | Mongolia | 2006 |
| IMAU20045 | ST4 | CC1 | 2 | 2 | 2 | 1 | 4 | 11 | 1 | 2 | 4 | 1 | 1  2  2  2  2  2  2  2  2  2  2  2  2  2  2  2  2  2  2  2  2  2  2  2  3  3  3  3  3  2  2  2  2  2  2  2  2  2  2  2  2  2  4  4  4  2  2  3  2  2  5  5  5  5  2  2  4  4  4  4  2  3  4  4  4  4  4  4  4  3  2  2  2  2  2  2  2  2  2  2  2  2  3  3  3  2  2  1  2  2  2  2  2  2  2  2  2  2  2  2  2  2  2  2  2  2  2  2  2  2  2  3  3  3  3  3  3  3  3  3  6  3  3  3  3  3  4  4  4  4  4  4  4  4  4  4  3  3  3  3  3  2  2  3  3  7  7  6  6  3  3  3  9  6  3  3  3  10  8  8  8  3  3  3  9  2  2  2  2  2  2  2  1  1  1 | Koumiss | Mongolia | 2006 |
| IMAU20046 | ST4 | CC1 | 2 | 2 | 2 | 1 | 4 | 11 | 1 | 2 | 4 | 1 | 1  2  2  2  2  2  2  2  2  2  2  2  2  2  2  2  2  2  2  2  2  2  2  2  3  3  3  3  3  2  2  2  2  2  2  2  2  2  2  2  2  2  4  4  4  2  2  3  2  2  5  5  5  5  2  2  4  4  4  4  2  3  4  4  4  4  4  4  4  3  2  2  2  2  2  2  2  2  2  2  2  2  3  3  3  2  2  1  2  2  2  2  2  2  2  2  2  2  2  2  2  2  2  2  2  2  2  2  2  2  2  3  3  3  3  3  3  3  3  3  6  3  3  3  3  3  4  4  4  4  4  4  4  4  4  4  3  3  3  3  3  2  2  3  3  7  7  6  6  3  3  3  9  6  3  3  3  10  8  8  8  3  3  3  9  2  2  2  2  2  2  2  1  1  1 | Koumiss | Mongolia | 2006 |
| IMAU20049 | ST4 | CC1 | 2 | 2 | 2 | 1 | 4 | 11 | 1 | 2 | 4 | 1 | 1  2  2  2  2  2  2  2  2  2  2  2  2  2  2  2  2  2  2  2  2  2  2  2  3  3  3  3  3  2  2  2  2  2  2  2  2  2  2  2  2  2  4  4  4  2  2  3  2  2  5  5  5  5  2  2  4  4  4  4  2  3  4  4  4  4  4  4  4  3  2  2  2  2  2  2  2  2  2  2  2  2  3  3  3  2  2  1  2  2  2  2  2  2  2  2  2  2  2  2  2  2  2  2  2  2  2  2  2  2  2  3  3  3  3  3  3  3  3  3  6  3  3  3  3  3  4  4  4  4  4  4  4  4  4  4  3  3  3  3  3  2  2  3  3  7  7  6  6  3  3  3  9  6  3  3  3  10  8  8  8  3  3  3  9  2  2  2  2  2  2  2  1  1  1 | Fermented camels’ milk | Mongolia | 2006 |
| IMAU20051 | ST4 | CC1 | 2 | 2 | 2 | 1 | 4 | 11 | 1 | 2 | 4 | 1 | 1  2  2  2  2  2  2  2  2  2  2  2  2  2  2  2  2  2  2  2  2  2  2  2  3  3  3  3  3  2  2  2  2  2  2  2  2  2  2  2  2  2  4  4  4  2  2  3  2  2  5  5  5  5  2  2  4  4  4  4  2  3  4  4  4  4  4  4  4  3  2  2  2  2  2  2  2  2  2  2  2  2  3  3  3  2  2  1  2  2  2  2  2  2  2  2  2  2  2  2  2  2  2  2  2  2  2  2  2  2  2  3  3  3  3  3  3  3  3  3  6  3  3  3  3  3  4  4  4  4  4  4  4  4  4  4  3  3  3  3  3  2  2  3  3  7  7  6  6  3  3  3  9  6  3  3  3  10  8  8  8  3  3  3  9  2  2  2  2  2  2  2  1  1  1 | Fermented camels’ milk | Mongolia | 2006 |
| IMAU20056 | ST4 | CC1 | 2 | 2 | 2 | 1 | 4 | 11 | 1 | 2 | 4 | 1 | 1  2  2  2  2  2  2  2  2  2  2  2  2  2  2  2  2  2  2  2  2  2  2  2  3  3  3  3  3  2  2  2  2  2  2  2  2  2  2  2  2  2  4  4  4  2  2  3  2  2  5  5  5  5  2  2  4  4  4  4  2  3  4  4  4  4  4  4  4  3  2  2  2  2  2  2  2  2  2  2  2  2  3  3  3  2  2  1  2  2  2  2  2  2  2  2  2  2  2  2  2  2  2  2  2  2  2  2  2  2  2  3  3  3  3  3  3  3  3  3  6  3  3  3  3  3  4  4  4  4  4  4  4  4  4  4  3  3  3  3  3  2  2  3  3  7  7  6  6  3  3  3  9  6  3  3  3  10  8  8  8  3  3  3  9  2  2  2  2  2  2  2  1  1  1 | Fermented camels’ milk | Mongolia | 2006 |
| IMAU20057 | ST4 | CC1 | 2 | 2 | 2 | 1 | 4 | 11 | 1 | 2 | 4 | 1 | 1  2  2  2  2  2  2  2  2  2  2  2  2  2  2  2  2  2  2  2  2  2  2  2  3  3  3  3  3  2  2  2  2  2  2  2  2  2  2  2  2  2  4  4  4  2  2  3  2  2  5  5  5  5  2  2  4  4  4  4  2  3  4  4  4  4  4  4  4  3  2  2  2  2  2  2  2  2  2  2  2  2  3  3  3  2  2  1  2  2  2  2  2  2  2  2  2  2  2  2  2  2  2  2  2  2  2  2  2  2  2  3  3  3  3  3  3  3  3  3  6  3  3  3  3  3  4  4  4  4  4  4  4  4  4  4  3  3  3  3  3  2  2  3  3  7  7  6  6  3  3  3  9  6  3  3  3  10  8  8  8  3  3  3  9  2  2  2  2  2  2  2  1  1  1 | Fermented camels’ milk | Mongolia | 2006 |
| IMAU20162 | ST4 | CC1 | 2 | 2 | 2 | 1 | 4 | 11 | 1 | 2 | 4 | 1 | 1  2  2  2  2  2  2  2  2  2  2  2  2  2  2  2  2  2  2  2  2  2  2  2  3  3  3  3  3  2  2  2  2  2  2  2  2  2  2  2  2  2  4  4  4  2  2  3  2  2  5  5  5  5  2  2  4  4  4  4  2  3  4  4  4  4  4  4  4  3  2  2  2  2  2  2  2  2  2  2  2  2  3  3  3  2  2  1  2  2  2  2  2  2  2  2  2  2  2  2  2  2  2  2  2  2  2  2  2  2  2  3  3  3  3  3  3  3  3  3  6  3  3  3  3  3  4  4  4  4  4  4  4  4  4  4  3  3  3  3  3  2  2  3  3  7  7  6  6  3  3  3  9  6  3  3  3  10  8  8  8  3  3  3  9  2  2  2  2  2  2  2  1  1  1 | Yoghurt | Mongolia | 2009 |
| IMAU20230 | ST4 | CC1 | 2 | 2 | 2 | 1 | 4 | 11 | 1 | 2 | 4 | 1 | 1  2  2  2  2  2  2  2  2  2  2  2  2  2  2  2  2  2  2  2  2  2  2  2  3  3  3  3  3  2  2  2  2  2  2  2  2  2  2  2  2  2  4  4  4  2  2  3  2  2  5  5  5  5  2  2  4  4  4  4  2  3  4  4  4  4  4  4  4  3  2  2  2  2  2  2  2  2  2  2  2  2  3  3  3  2  2  1  2  2  2  2  2  2  2  2  2  2  2  2  2  2  2  2  2  2  2  2  2  2  2  3  3  3  3  3  3  3  3  3  6  3  3  3  3  3  4  4  4  4  4  4  4  4  4  4  3  3  3  3  3  2  2  3  3  7  7  6  6  3  3  3  9  6  3  3  3  10  8  8  8  3  3  3  9  2  2  2  2  2  2  2  1  1  1 | Yoghurt | Mongolia | 2009 |
| IMAU20232 | ST4 | CC1 | 2 | 2 | 2 | 1 | 4 | 11 | 1 | 2 | 4 | 1 | 1  2  2  2  2  2  2  2  2  2  2  2  2  2  2  2  2  2  2  2  2  2  2  2  3  3  3  3  3  2  2  2  2  2  2  2  2  2  2  2  2  2  4  4  4  2  2  3  2  2  5  5  5  5  2  2  4  4  4  4  2  3  4  4  4  4  4  4  4  3  2  2  2  2  2  2  2  2  2  2  2  2  3  3  3  2  2  1  2  2  2  2  2  2  2  2  2  2  2  2  2  2  2  2  2  2  2  2  2  2  2  3  3  3  3  3  3  3  3  3  6  3  3  3  3  3  4  4  4  4  4  4  4  4  4  4  3  3  3  3  3  2  2  3  3  7  7  6  6  3  3  3  9  6  3  3  3  10  8  8  8  3  3  3  9  2  2  2  2  2  2  2  1  1  1 | Yoghurt | Mongolia | 2009 |
| IMAU20258 | ST4 | CC1 | 2 | 2 | 2 | 1 | 4 | 11 | 1 | 2 | 4 | 1 | 1  2  2  2  2  2  2  2  2  2  2  2  2  2  2  2  2  2  2  2  2  2  2  2  3  3  3  3  3  2  2  2  2  2  2  2  2  2  2  2  2  2  4  4  4  2  2  3  2  2  5  5  5  5  2  2  4  4  4  4  2  3  4  4  4  4  4  4  4  3  2  2  2  2  2  2  2  2  2  2  2  2  3  3  3  2  2  1  2  2  2  2  2  2  2  2  2  2  2  2  2  2  2  2  2  2  2  2  2  2  2  3  3  3  3  3  3  3  3  3  6  3  3  3  3  3  4  4  4  4  4  4  4  4  4  4  3  3  3  3  3  2  2  3  3  7  7  6  6  3  3  3  9  6  3  3  3  10  8  8  8  3  3  3  9  2  2  2  2  2  2  2  1  1  1 | Yoghurt | Mongolia | 2009 |
| IMAU20259 | ST4 | CC1 | 2 | 2 | 2 | 1 | 4 | 11 | 1 | 2 | 4 | 1 | 1  2  2  2  2  2  2  2  2  2  2  2  2  2  2  2  2  2  2  2  2  2  2  2  3  3  3  3  3  2  2  2  2  2  2  2  2  2  2  2  2  2  4  4  4  2  2  3  2  2  5  5  5  5  2  2  4  4  4  4  2  3  4  4  4  4  4  4  4  3  2  2  2  2  2  2  2  2  2  2  2  2  3  3  3  2  2  1  2  2  2  2  2  2  2  2  2  2  2  2  2  2  2  2  2  2  2  2  2  2  2  3  3  3  3  3  3  3  3  3  6  3  3  3  3  3  4  4  4  4  4  4  4  4  4  4  3  3  3  3  3  2  2  3  3  7  7  6  6  3  3  3  9  6  3  3  3  10  8  8  8  3  3  3  9  2  2  2  2  2  2  2  1  1  1 | Yoghurt | Mongolia | 2009 |
| IMAU20264 | ST4 | CC1 | 2 | 2 | 2 | 1 | 4 | 11 | 1 | 2 | 4 | 1 | 1  2  2  2  2  2  2  2  2  2  2  2  2  2  2  2  2  2  2  2  2  2  2  2  3  3  3  3  3  2  2  2  2  2  2  2  2  2  2  2  2  2  4  4  4  2  2  3  2  2  5  5  5  5  2  2  4  4  4  4  2  3  4  4  4  4  4  4  4  3  2  2  2  2  2  2  2  2  2  2  2  2  3  3  3  2  2  1  2  2  2  2  2  2  2  2  2  2  2  2  2  2  2  2  2  2  2  2  2  2  2  3  3  3  3  3  3  3  3  3  6  3  3  3  3  3  4  4  4  4  4  4  4  4  4  4  3  3  3  3  3  2  2  3  3  7  7  6  6  3  3  3  9  6  3  3  3  10  8  8  8  3  3  3  9  2  2  2  2  2  2  2  1  1  1 | Yoghurt | Mongolia | 2009 |
| IMAU20371 | ST4 | CC1 | 2 | 2 | 2 | 1 | 4 | 11 | 1 | 2 | 4 | 1 | 1  2  2  2  2  2  2  2  2  2  2  2  2  2  2  2  2  2  2  2  2  2  2  2  3  3  3  3  3  2  2  2  2  2  2  2  2  2  2  2  2  2  4  4  4  2  2  3  2  2  5  5  5  5  2  2  4  4  4  4  2  3  4  4  4  4  4  4  4  3  2  2  2  2  2  2  2  2  2  2  2  2  3  3  3  2  2  1  2  2  2  2  2  2  2  2  2  2  2  2  2  2  2  2  2  2  2  2  2  2  2  3  3  3  3  3  3  3  3  3  6  3  3  3  3  3  4  4  4  4  4  4  4  4  4  4  3  3  3  3  3  2  2  3  3  7  7  6  6  3  3  3  9  6  3  3  3  10  8  8  8  3  3  3  9  2  2  2  2  2  2  2  1  1  1 | Yoghurt | Mongolia | 2009 |
| IMAU20374 | ST4 | CC1 | 2 | 2 | 2 | 1 | 4 | 11 | 1 | 2 | 4 | 1 | 1  2  2  2  2  2  2  2  2  2  2  2  2  2  2  2  2  2  2  2  2  2  2  2  3  3  3  3  3  2  2  2  2  2  2  2  2  2  2  2  2  2  4  4  4  2  2  3  2  2  5  5  5  5  2  2  4  4  4  4  2  3  4  4  4  4  4  4  4  3  2  2  2  2  2  2  2  2  2  2  2  2  3  3  3  2  2  1  2  2  2  2  2  2  2  2  2  2  2  2  2  2  2  2  2  2  2  2  2  2  2  3  3  3  3  3  3  3  3  3  6  3  3  3  3  3  4  4  4  4  4  4  4  4  4  4  3  3  3  3  3  2  2  3  3  7  7  6  6  3  3  3  9  6  3  3  3  10  8  8  8  3  3  3  9  2  2  2  2  2  2  2  1  1  1 | Yoghurt | Mongolia | 2009 |
| IMAU20389 | ST4 | CC1 | 2 | 2 | 2 | 1 | 4 | 11 | 1 | 2 | 4 | 1 | 1  2  2  2  2  2  2  2  2  2  2  2  2  2  2  2  2  2  2  2  2  2  2  2  3  3  3  3  3  2  2  2  2  2  2  2  2  2  2  2  2  2  4  4  4  2  2  3  2  2  5  5  5  5  2  2  4  4  4  4  2  3  4  4  4  4  4  4  4  3  2  2  2  2  2  2  2  2  2  2  2  2  3  3  3  2  2  1  2  2  2  2  2  2  2  2  2  2  2  2  2  2  2  2  2  2  2  2  2  2  2  3  3  3  3  3  3  3  3  3  6  3  3  3  3  3  4  4  4  4  4  4  4  4  4  4  3  3  3  3  3  2  2  3  3  7  7  6  6  3  3  3  9  6  3  3  3  10  8  8  8  3  3  3  9  2  2  2  2  2  2  2  1  1  1 | Yoghurt | Mongolia | 2009 |
| IMAU20392 | ST4 | CC1 | 2 | 2 | 2 | 1 | 4 | 11 | 1 | 2 | 4 | 1 | 1  2  2  2  2  2  2  2  2  2  2  2  2  2  2  2  2  2  2  2  2  2  2  2  3  3  3  3  3  2  2  2  2  2  2  2  2  2  2  2  2  2  4  4  4  2  2  3  2  2  5  5  5  5  2  2  4  4  4  4  2  3  4  4  4  4  4  4  4  3  2  2  2  2  2  2  2  2  2  2  2  2  3  3  3  2  2  1  2  2  2  2  2  2  2  2  2  2  2  2  2  2  2  2  2  2  2  2  2  2  2  3  3  3  3  3  3  3  3  3  6  3  3  3  3  3  4  4  4  4  4  4  4  4  4  4  3  3  3  3  3  2  2  3  3  7  7  6  6  3  3  3  9  6  3  3  3  10  8  8  8  3  3  3  9  2  2  2  2  2  2  2  1  1  1 | Yoghurt | Mongolia | 2009 |
| IMAU20395 | ST4 | CC1 | 2 | 2 | 2 | 1 | 4 | 11 | 1 | 2 | 4 | 1 | 1  2  2  2  2  2  2  2  2  2  2  2  2  2  2  2  2  2  2  2  2  2  2  2  3  3  3  3  3  2  2  2  2  2  2  2  2  2  2  2  2  2  4  4  4  2  2  3  2  2  5  5  5  5  2  2  4  4  4  4  2  3  4  4  4  4  4  4  4  3  2  2  2  2  2  2  2  2  2  2  2  2  3  3  3  2  2  1  2  2  2  2  2  2  2  2  2  2  2  2  2  2  2  2  2  2  2  2  2  2  2  3  3  3  3  3  3  3  3  3  6  3  3  3  3  3  4  4  4  4  4  4  4  4  4  4  3  3  3  3  3  2  2  3  3  7  7  6  6  3  3  3  9  6  3  3  3  10  8  8  8  3  3  3  9  2  2  2  2  2  2  2  1  1  1 | Yoghurt | Mongolia | 2009 |
| IMAU70163 | ST4 | CC1 | 2 | 2 | 2 | 1 | 4 | 11 | 1 | 2 | 4 | 1 | 1  2  2  2  2  2  2  2  2  2  2  2  2  2  2  2  2  2  2  2  2  2  2  2  3  3  3  3  3  2  2  2  2  2  2  2  2  2  2  2  2  2  4  4  4  2  2  3  2  2  5  5  5  5  2  2  4  4  4  4  2  3  4  4  4  4  4  4  4  3  2  2  2  2  2  2  2  2  2  2  2  2  3  3  3  2  2  1  2  2  2  2  2  2  2  2  2  2  2  2  2  2  2  2  2  2  2  2  2  2  2  3  3  3  3  3  3  3  3  3  6  3  3  3  3  3  4  4  4  4  4  4  4  4  4  4  3  3  3  3  3  2  2  3  3  7  7  6  6  3  3  3  9  6  3  3  3  10  8  8  8  3  3  3  9  2  2  2  2  2  2  2  1  1  1 | Acidic gruel | Inner Mongolia | 2008 |
| IMAU20060 | ST4 | CC1 | 2 | 2 | 2 | 1 | 4 | 11 | 1 | 2 | 4 | 1 | 1  2  2  2  2  2  2  2  2  2  2  2  2  2  2  2  2  2  2  2  2  2  2  2  3  3  3  3  3  2  2  2  2  2  2  2  2  2  2  2  2  2  4  4  4  2  2  3  2  2  5  5  5  5  2  2  4  4  4  4  2  3  4  4  4  4  4  4  4  3  2  2  2  2  2  2  2  2  2  2  2  2  3  3  3  2  2  1  2  2  2  2  2  2  2  2  2  2  2  2  2  2  2  2  2  2  2  2  2  2  2  3  3  3  3  3  3  3  3  3  6  3  3  3  3  3  4  4  4  4  4  4  4  4  4  4  3  3  3  3  3  2  2  3  3  7  7  6  6  3  3  3  9  6  3  3  3  10  8  8  8  3  3  3  9  2  2  2  2  2  2  2  1  1  1 | Fermented camels’ milk | Mongolia | 2006 |
| IMAU40112 | ST4 | CC1 | 2 | 2 | 2 | 1 | 4 | 11 | 1 | 2 | 4 | 1 | 1  2  2  2  2  2  2  2  2  2  2  2  2  2  2  2  2  2  2  2  2  2  2  2  3  3  3  3  3  2  2  2  2  2  2  2  2  2  2  2  2  2  4  4  4  2  2  3  2  2  5  5  5  5  2  2  4  4  4  4  2  3  4  4  4  4  4  4  4  3  2  2  2  2  2  2  2  2  2  2  2  2  3  3  3  2  2  1  2  2  2  2  2  2  2  2  2  2  2  2  2  2  2  2  2  2  2  2  2  2  2  3  3  3  3  3  3  3  3  3  6  3  3  3  3  3  4  4  4  4  4  4  4  4  4  4  3  3  3  3  3  2  2  3  3  7  7  6  6  3  3  3  9  6  3  3  3  10  8  8  8  3  3  3  9  2  2  2  2  2  2  2  1  1  1 | Kurut | Qinghai | 2005 |
| IMAU40124 | ST4 | CC1 | 2 | 2 | 2 | 1 | 4 | 11 | 1 | 2 | 4 | 1 | 1  2  2  2  2  2  2  2  2  2  2  2  2  2  2  2  2  2  2  2  2  2  2  2  3  3  3  3  3  2  2  2  2  2  2  2  2  2  2  2  2  2  4  4  4  2  2  3  2  2  5  5  5  5  2  2  4  4  4  4  2  3  4  4  4  4  4  4  4  3  2  2  2  2  2  2  2  2  2  2  2  2  3  3  3  2  2  1  2  2  2  2  2  2  2  2  2  2  2  2  2  2  2  2  2  2  2  2  2  2  2  3  3  3  3  3  3  3  3  3  6  3  3  3  3  3  4  4  4  4  4  4  4  4  4  4  3  3  3  3  3  2  2  3  3  7  7  6  6  3  3  3  9  6  3  3  3  10  8  8  8  3  3  3  9  2  2  2  2  2  2  2  1  1  1 | Kurut | Qinghai | 2005 |
| IMAU80316 | ST4 | CC1 | 2 | 2 | 2 | 1 | 4 | 11 | 1 | 2 | 4 | 1 | 1  2  2  2  2  2  2  2  2  2  2  2  2  2  2  2  2  2  2  2  2  2  2  2  3  3  3  3  3  2  2  2  2  2  2  2  2  2  2  2  2  2  4  4  4  2  2  3  2  2  5  5  5  5  2  2  4  4  4  4  2  3  4  4  4  4  4  4  4  3  2  2  2  2  2  2  2  2  2  2  2  2  3  3  3  2  2  1  2  2  2  2  2  2  2  2  2  2  2  2  2  2  2  2  2  2  2  2  2  2  2  3  3  3  3  3  3  3  3  3  6  3  3  3  3  3  4  4  4  4  4  4  4  4  4  4  3  3  3  3  3  2  2  3  3  7  7  6  6  3  3  3  9  6  3  3  3  10  8  8  8  3  3  3  9  2  2  2  2  2  2  2  1  1  1 | Whey | Sichuan | 2009 |
| IMAU80364 | ST4 | CC1 | 2 | 2 | 2 | 1 | 4 | 11 | 1 | 2 | 4 | 1 | 1  2  2  2  2  2  2  2  2  2  2  2  2  2  2  2  2  2  2  2  2  2  2  2  3  3  3  3  3  2  2  2  2  2  2  2  2  2  2  2  2  2  4  4  4  2  2  3  2  2  5  5  5  5  2  2  4  4  4  4  2  3  4  4  4  4  4  4  4  3  2  2  2  2  2  2  2  2  2  2  2  2  3  3  3  2  2  1  2  2  2  2  2  2  2  2  2  2  2  2  2  2  2  2  2  2  2  2  2  2  2  3  3  3  3  3  3  3  3  3  6  3  3  3  3  3  4  4  4  4  4  4  4  4  4  4  3  3  3  3  3  2  2  3  3  7  7  6  6  3  3  3  9  6  3  3  3  10  8  8  8  3  3  3  9  2  2  2  2  2  2  2  1  1  1 | Qula | Sichuan | 2009 |
| IMAU80566 | ST4 | CC1 | 2 | 2 | 2 | 1 | 4 | 11 | 1 | 2 | 4 | 1 | 1  2  2  2  2  2  2  2  2  2  2  2  2  2  2  2  2  2  2  2  2  2  2  2  3  3  3  3  3  2  2  2  2  2  2  2  2  2  2  2  2  2  4  4  4  2  2  3  2  2  5  5  5  5  2  2  4  4  4  4  2  3  4  4  4  4  4  4  4  3  2  2  2  2  2  2  2  2  2  2  2  2  3  3  3  2  2  1  2  2  2  2  2  2  2  2  2  2  2  2  2  2  2  2  2  2  2  2  2  2  2  3  3  3  3  3  3  3  3  3  6  3  3  3  3  3  4  4  4  4  4  4  4  4  4  4  3  3  3  3  3  2  2  3  3  7  7  6  6  3  3  3  9  6  3  3  3  10  8  8  8  3  3  3  9  2  2  2  2  2  2  2  1  1  1 | Cream | Gansu | 2009 |
| IMAU80571 | ST4 | CC1 | 2 | 2 | 2 | 1 | 4 | 11 | 1 | 2 | 4 | 1 | 1  2  2  2  2  2  2  2  2  2  2  2  2  2  2  2  2  2  2  2  2  2  2  2  3  3  3  3  3  2  2  2  2  2  2  2  2  2  2  2  2  2  4  4  4  2  2  3  2  2  5  5  5  5  2  2  4  4  4  4  2  3  4  4  4  4  4  4  4  3  2  2  2  2  2  2  2  2  2  2  2  2  3  3  3  2  2  1  2  2  2  2  2  2  2  2  2  2  2  2  2  2  2  2  2  2  2  2  2  2  2  3  3  3  3  3  3  3  3  3  6  3  3  3  3  3  4  4  4  4  4  4  4  4  4  4  3  3  3  3  3  2  2  3  3  7  7  6  6  3  3  3  9  6  3  3  3  10  8  8  8  3  3  3  9  2  2  2  2  2  2  2  1  1  1 | Kurut | Gansu | 2009 |
| IMAU80607 | ST4 | CC1 | 2 | 2 | 2 | 1 | 4 | 11 | 1 | 2 | 4 | 1 | 1  2  2  2  2  2  2  2  2  2  2  2  2  2  2  2  2  2  2  2  2  2  2  2  3  3  3  3  3  2  2  2  2  2  2  2  2  2  2  2  2  2  4  4  4  2  2  3  2  2  5  5  5  5  2  2  4  4  4  4  2  3  4  4  4  4  4  4  4  3  2  2  2  2  2  2  2  2  2  2  2  2  3  3  3  2  2  1  2  2  2  2  2  2  2  2  2  2  2  2  2  2  2  2  2  2  2  2  2  2  2  3  3  3  3  3  3  3  3  3  6  3  3  3  3  3  4  4  4  4  4  4  4  4  4  4  3  3  3  3  3  2  2  3  3  7  7  6  6  3  3  3  9  6  3  3  3  10  8  8  8  3  3  3  9  2  2  2  2  2  2  2  1  1  1 | Yoghurt | Gansu | 2009 |
| IMAU80609 | ST4 | CC1 | 2 | 2 | 2 | 1 | 4 | 11 | 1 | 2 | 4 | 1 | 1  2  2  2  2  2  2  2  2  2  2  2  2  2  2  2  2  2  2  2  2  2  2  2  3  3  3  3  3  2  2  2  2  2  2  2  2  2  2  2  2  2  4  4  4  2  2  3  2  2  5  5  5  5  2  2  4  4  4  4  2  3  4  4  4  4  4  4  4  3  2  2  2  2  2  2  2  2  2  2  2  2  3  3  3  2  2  1  2  2  2  2  2  2  2  2  2  2  2  2  2  2  2  2  2  2  2  2  2  2  2  3  3  3  3  3  3  3  3  3  6  3  3  3  3  3  4  4  4  4  4  4  4  4  4  4  3  3  3  3  3  2  2  3  3  7  7  6  6  3  3  3  9  6  3  3  3  10  8  8  8  3  3  3  9  2  2  2  2  2  2  2  1  1  1 | Qula | Gansu | 2009 |
| IMAU20064 | ST4 | CC1 | 2 | 2 | 2 | 1 | 4 | 11 | 1 | 2 | 4 | 1 | 1  2  2  2  2  2  2  2  2  2  2  2  2  2  2  2  2  2  2  2  2  2  2  2  3  3  3  3  3  2  2  2  2  2  2  2  2  2  2  2  2  2  4  4  4  2  2  3  2  2  5  5  5  5  2  2  4  4  4  4  2  3  4  4  4  4  4  4  4  3  2  2  2  2  2  2  2  2  2  2  2  2  3  3  3  2  2  1  2  2  2  2  2  2  2  2  2  2  2  2  2  2  2  2  2  2  2  2  2  2  2  3  3  3  3  3  3  3  3  3  6  3  3  3  3  3  4  4  4  4  4  4  4  4  4  4  3  3  3  3  3  2  2  3  3  7  7  6  6  3  3  3  9  6  3  3  3  10  8  8  8  3  3  3  9  2  2  2  2  2  2  2  1  1  1 | Fermented camels’ milk | Mongolia | 2006 |
| IMAU60046 | ST4 | CC1 | 2 | 2 | 2 | 1 | 4 | 11 | 1 | 2 | 4 | 1 | 1  2  2  2  2  2  2  2  2  2  2  2  2  2  2  2  2  2  2  2  2  2  2  2  3  3  3  3  3  2  2  2  2  2  2  2  2  2  2  2  2  2  4  4  4  2  2  3  2  2  5  5  5  5  2  2  4  4  4  4  2  3  4  4  4  4  4  4  4  3  2  2  2  2  2  2  2  2  2  2  2  2  3  3  3  2  2  1  2  2  2  2  2  2  2  2  2  2  2  2  2  2  2  2  2  2  2  2  2  2  2  3  3  3  3  3  3  3  3  3  6  3  3  3  3  3  4  4  4  4  4  4  4  4  4  4  3  3  3  3  3  2  2  3  3  7  7  6  6  3  3  3  9  6  3  3  3  10  8  8  8  3  3  3  9  2  2  2  2  2  2  2  1  1  1 | Yoghurt | Tibet | 2007 |
| IMAU60070 | ST4 | CC1 | 2 | 2 | 2 | 1 | 4 | 11 | 1 | 2 | 4 | 1 | 1  2  2  2  2  2  2  2  2  2  2  2  2  2  2  2  2  2  2  2  2  2  2  2  3  3  3  3  3  2  2  2  2  2  2  2  2  2  2  2  2  2  4  4  4  2  2  3  2  2  5  5  5  5  2  2  4  4  4  4  2  3  4  4  4  4  4  4  4  3  2  2  2  2  2  2  2  2  2  2  2  2  3  3  3  2  2  1  2  2  2  2  2  2  2  2  2  2  2  2  2  2  2  2  2  2  2  2  2  2  2  3  3  3  3  3  3  3  3  3  6  3  3  3  3  3  4  4  4  4  4  4  4  4  4  4  3  3  3  3  3  2  2  3  3  7  7  6  6  3  3  3  9  6  3  3  3  10  8  8  8  3  3  3  9  2  2  2  2  2  2  2  1  1  1 | Kurut | Tibet | 2007 |
| IMAU60086 | ST4 | CC1 | 2 | 2 | 2 | 1 | 4 | 11 | 1 | 2 | 4 | 1 | 1  2  2  2  2  2  2  2  2  2  2  2  2  2  2  2  2  2  2  2  2  2  2  2  3  3  3  3  3  2  2  2  2  2  2  2  2  2  2  2  2  2  4  4  4  2  2  3  2  2  5  5  5  5  2  2  4  4  4  4  2  3  4  4  4  4  4  4  4  3  2  2  2  2  2  2  2  2  2  2  2  2  3  3  3  2  2  1  2  2  2  2  2  2  2  2  2  2  2  2  2  2  2  2  2  2  2  2  2  2  2  3  3  3  3  3  3  3  3  3  6  3  3  3  3  3  4  4  4  4  4  4  4  4  4  4  3  3  3  3  3  2  2  3  3  7  7  6  6  3  3  3  9  6  3  3  3  10  8  8  8  3  3  3  9  2  2  2  2  2  2  2  1  1  1 | Kurut | Tibet | 2007 |
| IMAU60121 | ST4 | CC1 | 2 | 2 | 2 | 1 | 4 | 11 | 1 | 2 | 4 | 1 | 1  2  2  2  2  2  2  2  2  2  2  2  2  2  2  2  2  2  2  2  2  2  2  2  3  3  3  3  3  2  2  2  2  2  2  2  2  2  2  2  2  2  4  4  4  2  2  3  2  2  5  5  5  5  2  2  4  4  4  4  2  3  4  4  4  4  4  4  4  3  2  2  2  2  2  2  2  2  2  2  2  2  3  3  3  2  2  1  2  2  2  2  2  2  2  2  2  2  2  2  2  2  2  2  2  2  2  2  2  2  2  3  3  3  3  3  3  3  3  3  6  3  3  3  3  3  4  4  4  4  4  4  4  4  4  4  3  3  3  3  3  2  2  3  3  7  7  6  6  3  3  3  9  6  3  3  3  10  8  8  8  3  3  3  9  2  2  2  2  2  2  2  1  1  1 | Kurut | Tibet | 2007 |
| IMAU60140 | ST4 | CC1 | 2 | 2 | 2 | 1 | 4 | 11 | 1 | 2 | 4 | 1 | 1  2  2  2  2  2  2  2  2  2  2  2  2  2  2  2  2  2  2  2  2  2  2  2  3  3  3  3  3  2  2  2  2  2  2  2  2  2  2  2  2  2  4  4  4  2  2  3  2  2  5  5  5  5  2  2  4  4  4  4  2  3  4  4  4  4  4  4  4  3  2  2  2  2  2  2  2  2  2  2  2  2  3  3  3  2  2  1  2  2  2  2  2  2  2  2  2  2  2  2  2  2  2  2  2  2  2  2  2  2  2  3  3  3  3  3  3  3  3  3  6  3  3  3  3  3  4  4  4  4  4  4  4  4  4  4  3  3  3  3  3  2  2  3  3  7  7  6  6  3  3  3  9  6  3  3  3  10  8  8  8  3  3  3  9  2  2  2  2  2  2  2  1  1  1 | Kurut | Tibet | 2007 |
| IMAU60146 | ST4 | CC1 | 2 | 2 | 2 | 1 | 4 | 11 | 1 | 2 | 4 | 1 | 1  2  2  2  2  2  2  2  2  2  2  2  2  2  2  2  2  2  2  2  2  2  2  2  3  3  3  3  3  2  2  2  2  2  2  2  2  2  2  2  2  2  4  4  4  2  2  3  2  2  5  5  5  5  2  2  4  4  4  4  2  3  4  4  4  4  4  4  4  3  2  2  2  2  2  2  2  2  2  2  2  2  3  3  3  2  2  1  2  2  2  2  2  2  2  2  2  2  2  2  2  2  2  2  2  2  2  2  2  2  2  3  3  3  3  3  3  3  3  3  6  3  3  3  3  3  4  4  4  4  4  4  4  4  4  4  3  3  3  3  3  2  2  3  3  7  7  6  6  3  3  3  9  6  3  3  3  10  8  8  8  3  3  3  9  2  2  2  2  2  2  2  1  1  1 | Kurut | Tibet | 2007 |
| IMAU60149 | ST4 | CC1 | 2 | 2 | 2 | 1 | 4 | 11 | 1 | 2 | 4 | 1 | 1  2  2  2  2  2  2  2  2  2  2  2  2  2  2  2  2  2  2  2  2  2  2  2  3  3  3  3  3  2  2  2  2  2  2  2  2  2  2  2  2  2  4  4  4  2  2  3  2  2  5  5  5  5  2  2  4  4  4  4  2  3  4  4  4  4  4  4  4  3  2  2  2  2  2  2  2  2  2  2  2  2  3  3  3  2  2  1  2  2  2  2  2  2  2  2  2  2  2  2  2  2  2  2  2  2  2  2  2  2  2  3  3  3  3  3  3  3  3  3  6  3  3  3  3  3  4  4  4  4  4  4  4  4  4  4  3  3  3  3  3  2  2  3  3  7  7  6  6  3  3  3  9  6  3  3  3  10  8  8  8  3  3  3  9  2  2  2  2  2  2  2  1  1  1 | Kurut | Tibet | 2007 |
| IMAU20653 | ST4 | CC1 | 2 | 2 | 2 | 1 | 4 | 11 | 1 | 2 | 4 | 1 | 1  2  2  2  2  2  2  2  2  2  2  2  2  2  2  2  2  2  2  2  2  2  2  2  3  3  3  3  3  2  2  2  2  2  2  2  2  2  2  2  2  2  4  4  4  2  2  3  2  2  5  5  5  5  2  2  4  4  4  4  2  3  4  4  4  4  4  4  4  3  2  2  2  2  2  2  2  2  2  2  2  2  3  3  3  2  2  1  2  2  2  2  2  2  2  2  2  2  2  2  2  2  2  2  2  2  2  2  2  2  2  3  3  3  3  3  3  3  3  3  6  3  3  3  3  3  4  4  4  4  4  4  4  4  4  4  3  3  3  3  3  2  2  3  3  7  7  6  6  3  3  3  9  6  3  3  3  10  8  8  8  3  3  3  9  2  2  2  2  2  2  2  1  1  1 | Yoghurt | Mongolia | 2009 |
| IMAU80304 | ST4 | CC1 | 2 | 2 | 2 | 1 | 4 | 11 | 1 | 2 | 4 | 1 | 1  2  2  2  2  2  2  2  2  2  2  2  2  2  2  2  2  2  2  2  2  2  2  2  3  3  3  3  3  2  2  2  2  2  2  2  2  2  2  2  2  2  4  4  4  2  2  3  2  2  5  5  5  5  2  2  4  4  4  4  2  3  4  4  4  4  4  4  4  3  2  2  2  2  2  2  2  2  2  2  2  2  3  3  3  2  2  1  2  2  2  2  2  2  2  2  2  2  2  2  2  2  2  2  2  2  2  2  2  2  2  3  3  3  3  3  3  3  3  3  6  3  3  3  3  3  4  4  4  4  4  4  4  4  4  4  3  3  3  3  3  2  2  3  3  7  7  6  6  3  3  3  9  6  3  3  3  10  8  8  8  3  3  3  9  2  2  2  2  2  2  2  1  1  1 | Qula | Sichuan | 2009 |
| IMAU60104 | ST4 | CC1 | 2 | 2 | 2 | 1 | 4 | 11 | 1 | 2 | 4 | 1 | 1  2  2  2  2  2  2  2  2  2  2  2  2  2  2  2  2  2  2  2  2  2  2  2  3  3  3  3  3  2  2  2  2  2  2  2  2  2  2  2  2  2  4  4  4  2  2  3  2  2  5  5  5  5  2  2  4  4  4  4  2  3  4  4  4  4  4  4  4  3  2  2  2  2  2  2  2  2  2  2  2  2  3  3  3  2  2  1  2  2  2  2  2  2  2  2  2  2  2  2  2  2  2  2  2  2  2  2  2  2  2  3  3  3  3  3  3  3  3  3  6  3  3  3  3  3  4  4  4  4  4  4  4  4  4  4  3  3  3  3  3  2  2  3  3  7  7  6  6  3  3  3  9  6  3  3  3  10  8  8  8  3  3  3  9  2  2  2  2  2  2  2  1  1  1 | Kurut | Tibet | 2007 |
| IMAU20037 | ST5 | CC1 | 2 | 2 | 2 | 1 | 4 | 11 | 3 | 2 | 4 | 1 | 1  2  2  2  2  2  2  2  2  2  2  2  2  2  2  2  2  2  2  2  2  2  2  2  3  3  3  3  3  2  2  2  2  2  2  2  2  2  2  2  2  2  4  4  4  2  2  3  2  2  5  5  5  5  2  2  4  4  4  4  2  3  4  4  4  4  4  4  4  3  2  2  2  2  2  2  2  2  2  2  2  2  3  3  3  2  2  1  2  2  2  2  2  2  2  2  2  2  2  2  2  2  2  2  2  2  2  2  2  2  2  3  3  3  3  3  3  3  3  3  6  3  3  3  3  3  4  4  4  4  4  4  4  4  4  4  3  3  3  3  3  2  2  3  3  7  7  6  6  3  3  3  9  6  3  3  3  10  8  8  8  3  3  3  9  2  2  2  2  2  2  2  1  1  1 | Fermented camels’ milk | Mongolia | 2006 |
| IMAU20052 | ST5 | CC1 | 2 | 2 | 2 | 1 | 4 | 11 | 3 | 2 | 4 | 1 | 1  2  2  2  2  2  2  2  2  2  2  2  2  2  2  2  2  2  2  2  2  2  2  2  3  3  3  3  3  2  2  2  2  2  2  2  2  2  2  2  2  2  4  4  4  2  2  3  2  2  5  5  5  5  2  2  4  4  4  4  2  3  4  4  4  4  4  4  4  3  2  2  2  2  2  2  2  2  2  2  2  2  3  3  3  2  2  1  2  2  2  2  2  2  2  2  2  2  2  2  2  2  2  2  2  2  2  2  2  2  2  3  3  3  3  3  3  3  3  3  6  3  3  3  3  3  4  4  4  4  4  4  4  4  4  4  3  3  3  3  3  2  2  3  3  7  7  6  6  3  3  3  9  6  3  3  3  10  8  8  8  3  3  3  9  2  2  2  2  2  2  2  1  1  1 | Fermented camels’ milk | Mongolia | 2006 |
| IMAU20054 | ST5 | CC1 | 2 | 2 | 2 | 1 | 4 | 11 | 3 | 2 | 4 | 1 | 1  2  2  2  2  2  2  2  2  2  2  2  2  2  2  2  2  2  2  2  2  2  2  2  3  3  3  3  3  2  2  2  2  2  2  2  2  2  2  2  2  2  4  4  4  2  2  3  2  2  5  5  5  5  2  2  4  4  4  4  2  3  4  4  4  4  4  4  4  3  2  2  2  2  2  2  2  2  2  2  2  2  3  3  3  2  2  1  2  2  2  2  2  2  2  2  2  2  2  2  2  2  2  2  2  2  2  2  2  2  2  3  3  3  3  3  3  3  3  3  6  3  3  3  3  3  4  4  4  4  4  4  4  4  4  4  3  3  3  3  3  2  2  3  3  7  7  6  6  3  3  3  9  6  3  3  3  10  8  8  8  3  3  3  9  2  2  2  2  2  2  2  1  1  1 | Fermented camels’ milk | Mongolia | 2006 |
| IMAU20055 | ST5 | CC1 | 2 | 2 | 2 | 1 | 4 | 11 | 3 | 2 | 4 | 1 | 1  2  2  2  2  2  2  2  2  2  2  2  2  2  2  2  2  2  2  2  2  2  2  2  3  3  3  3  3  2  2  2  2  2  2  2  2  2  2  2  2  2  4  4  4  2  2  3  2  2  5  5  5  5  2  2  4  4  4  4  2  3  4  4  4  4  4  4  4  3  2  2  2  2  2  2  2  2  2  2  2  2  3  3  3  2  2  1  2  2  2  2  2  2  2  2  2  2  2  2  2  2  2  2  2  2  2  2  2  2  2  3  3  3  3  3  3  3  3  3  6  3  3  3  3  3  4  4  4  4  4  4  4  4  4  4  3  3  3  3  3  2  2  3  3  7  7  6  6  3  3  3  9  6  3  3  3  10  8  8  8  3  3  3  9  2  2  2  2  2  2  2  1  1  1 | Fermented camels’ milk | Mongolia | 2006 |
| IMAU20059 | ST5 | CC1 | 2 | 2 | 2 | 1 | 4 | 11 | 3 | 2 | 4 | 1 | 1  2  2  2  2  2  2  2  2  2  2  2  2  2  2  2  2  2  2  2  2  2  2  2  3  3  3  3  3  2  2  2  2  2  2  2  2  2  2  2  2  2  4  4  4  2  2  3  2  2  5  5  5  5  2  2  4  4  4  4  2  3  4  4  4  4  4  4  4  3  2  2  2  2  2  2  2  2  2  2  2  2  3  3  3  2  2  1  2  2  2  2  2  2  2  2  2  2  2  2  2  2  2  2  2  2  2  2  2  2  2  3  3  3  3  3  3  3  3  3  6  3  3  3  3  3  4  4  4  4  4  4  4  4  4  4  3  3  3  3  3  2  2  3  3  7  7  6  6  3  3  3  9  6  3  3  3  10  8  8  8  3  3  3  9  2  2  2  2  2  2  2  1  1  1 | Fermented camels’ milk | Mongolia | 2006 |
| IMAU20065 | ST5 | CC1 | 2 | 2 | 2 | 1 | 4 | 11 | 3 | 2 | 4 | 1 | 1  2  2  2  2  2  2  2  2  2  2  2  2  2  2  2  2  2  2  2  2  2  2  2  3  3  3  3  3  2  2  2  2  2  2  2  2  2  2  2  2  2  4  4  4  2  2  3  2  2  5  5  5  5  2  2  4  4  4  4  2  3  4  4  4  4  4  4  4  3  2  2  2  2  2  2  2  2  2  2  2  2  3  3  3  2  2  1  2  2  2  2  2  2  2  2  2  2  2  2  2  2  2  2  2  2  2  2  2  2  2  3  3  3  3  3  3  3  3  3  6  3  3  3  3  3  4  4  4  4  4  4  4  4  4  4  3  3  3  3  3  2  2  3  3  7  7  6  6  3  3  3  9  6  3  3  3  10  8  8  8  3  3  3  9  2  2  2  2  2  2  2  1  1  1 | Fermented camels’ milk | Mongolia | 2006 |
| IMAU20079 | ST5 | CC1 | 2 | 2 | 2 | 1 | 4 | 11 | 3 | 2 | 4 | 1 | 1  2  2  2  2  2  2  2  2  2  2  2  2  2  2  2  2  2  2  2  2  2  2  2  3  3  3  3  3  2  2  2  2  2  2  2  2  2  2  2  2  2  4  4  4  2  2  3  2  2  5  5  5  5  2  2  4  4  4  4  2  3  4  4  4  4  4  4  4  3  2  2  2  2  2  2  2  2  2  2  2  2  3  3  3  2  2  1  2  2  2  2  2  2  2  2  2  2  2  2  2  2  2  2  2  2  2  2  2  2  2  3  3  3  3  3  3  3  3  3  6  3  3  3  3  3  4  4  4  4  4  4  4  4  4  4  3  3  3  3  3  2  2  3  3  7  7  6  6  3  3  3  9  6  3  3  3  10  8  8  8  3  3  3  9  2  2  2  2  2  2  2  1  1  1 | Fermented camels’ milk | Mongolia | 2006 |
| IMAU20081 | ST5 | CC1 | 2 | 2 | 2 | 1 | 4 | 11 | 3 | 2 | 4 | 1 | 1  2  2  2  2  2  2  2  2  2  2  2  2  2  2  2  2  2  2  2  2  2  2  2  3  3  3  3  3  2  2  2  2  2  2  2  2  2  2  2  2  2  4  4  4  2  2  3  2  2  5  5  5  5  2  2  4  4  4  4  2  3  4  4  4  4  4  4  4  3  2  2  2  2  2  2  2  2  2  2  2  2  3  3  3  2  2  1  2  2  2  2  2  2  2  2  2  2  2  2  2  2  2  2  2  2  2  2  2  2  2  3  3  3  3  3  3  3  3  3  6  3  3  3  3  3  4  4  4  4  4  4  4  4  4  4  3  3  3  3  3  2  2  3  3  7  7  6  6  3  3  3  9  6  3  3  3  10  8  8  8  3  3  3  9  2  2  2  2  2  2  2  1  1  1 | Fermented goats’ milk | Mongolia | 2006 |
| IMAU20083 | ST5 | CC1 | 2 | 2 | 2 | 1 | 4 | 11 | 3 | 2 | 4 | 1 | 1  2  2  2  2  2  2  2  2  2  2  2  2  2  2  2  2  2  2  2  2  2  2  2  3  3  3  3  3  2  2  2  2  2  2  2  2  2  2  2  2  2  4  4  4  2  2  3  2  2  5  5  5  5  2  2  4  4  4  4  2  3  4  4  4  4  4  4  4  3  2  2  2  2  2  2  2  2  2  2  2  2  3  3  3  2  2  1  2  2  2  2  2  2  2  2  2  2  2  2  2  2  2  2  2  2  2  2  2  2  2  3  3  3  3  3  3  3  3  3  6  3  3  3  3  3  4  4  4  4  4  4  4  4  4  4  3  3  3  3  3  2  2  3  3  7  7  6  6  3  3  3  9  6  3  3  3  10  8  8  8  3  3  3  9  2  2  2  2  2  2  2  1  1  1 | Fermented camels’ milk | Mongolia | 2006 |
| IMAU20085 | ST5 | CC1 | 2 | 2 | 2 | 1 | 4 | 11 | 3 | 2 | 4 | 1 | 1  2  2  2  2  2  2  2  2  2  2  2  2  2  2  2  2  2  2  2  2  2  2  2  3  3  3  3  3  2  2  2  2  2  2  2  2  2  2  2  2  2  4  4  4  2  2  3  2  2  5  5  5  5  2  2  4  4  4  4  2  3  4  4  4  4  4  4  4  3  2  2  2  2  2  2  2  2  2  2  2  2  3  3  3  2  2  1  2  2  2  2  2  2  2  2  2  2  2  2  2  2  2  2  2  2  2  2  2  2  2  3  3  3  3  3  3  3  3  3  6  3  3  3  3  3  4  4  4  4  4  4  4  4  4  4  3  3  3  3  3  2  2  3  3  7  7  6  6  3  3  3  9  6  3  3  3  10  8  8  8  3  3  3  9  2  2  2  2  2  2  2  1  1  1 | Fermented camels’ milk | Mongolia | 2006 |
| IMAU20086 | ST5 | CC1 | 2 | 2 | 2 | 1 | 4 | 11 | 3 | 2 | 4 | 1 | 1  2  2  2  2  2  2  2  2  2  2  2  2  2  2  2  2  2  2  2  2  2  2  2  3  3  3  3  3  2  2  2  2  2  2  2  2  2  2  2  2  2  4  4  4  2  2  3  2  2  5  5  5  5  2  2  4  4  4  4  2  3  4  4  4  4  4  4  4  3  2  2  2  2  2  2  2  2  2  2  2  2  3  3  3  2  2  1  2  2  2  2  2  2  2  2  2  2  2  2  2  2  2  2  2  2  2  2  2  2  2  3  3  3  3  3  3  3  3  3  6  3  3  3  3  3  4  4  4  4  4  4  4  4  4  4  3  3  3  3  3  2  2  3  3  7  7  6  6  3  3  3  9  6  3  3  3  10  8  8  8  3  3  3  9  2  2  2  2  2  2  2  1  1  1 | Fermented camels’ milk | Mongolia | 2006 |
| IMAU20087 | ST5 | CC1 | 2 | 2 | 2 | 1 | 4 | 11 | 3 | 2 | 4 | 1 | 1  2  2  2  2  2  2  2  2  2  2  2  2  2  2  2  2  2  2  2  2  2  2  2  3  3  3  3  3  2  2  2  2  2  2  2  2  2  2  2  2  2  4  4  4  2  2  3  2  2  5  5  5  5  2  2  4  4  4  4  2  3  4  4  4  4  4  4  4  3  2  2  2  2  2  2  2  2  2  2  2  2  3  3  3  2  2  1  2  2  2  2  2  2  2  2  2  2  2  2  2  2  2  2  2  2  2  2  2  2  2  3  3  3  3  3  3  3  3  3  6  3  3  3  3  3  4  4  4  4  4  4  4  4  4  4  3  3  3  3  3  2  2  3  3  7  7  6  6  3  3  3  9  6  3  3  3  10  8  8  8  3  3  3  9  2  2  2  2  2  2  2  1  1  1 | Fermented camels’ milk | Mongolia | 2006 |
| IMAU20088 | ST5 | CC1 | 2 | 2 | 2 | 1 | 4 | 11 | 3 | 2 | 4 | 1 | 1  2  2  2  2  2  2  2  2  2  2  2  2  2  2  2  2  2  2  2  2  2  2  2  3  3  3  3  3  2  2  2  2  2  2  2  2  2  2  2  2  2  4  4  4  2  2  3  2  2  5  5  5  5  2  2  4  4  4  4  2  3  4  4  4  4  4  4  4  3  2  2  2  2  2  2  2  2  2  2  2  2  3  3  3  2  2  1  2  2  2  2  2  2  2  2  2  2  2  2  2  2  2  2  2  2  2  2  2  2  2  3  3  3  3  3  3  3  3  3  6  3  3  3  3  3  4  4  4  4  4  4  4  4  4  4  3  3  3  3  3  2  2  3  3  7  7  6  6  3  3  3  9  6  3  3  3  10  8  8  8  3  3  3  9  2  2  2  2  2  2  2  1  1  1 | Fermented camels’ milk | Mongolia | 2006 |
| IMAU20315 | ST5 | CC1 | 2 | 2 | 2 | 1 | 4 | 11 | 3 | 2 | 4 | 1 | 1  2  2  2  2  2  2  2  2  2  2  2  2  2  2  2  2  2  2  2  2  2  2  2  3  3  3  3  3  2  2  2  2  2  2  2  2  2  2  2  2  2  4  4  4  2  2  3  2  2  5  5  5  5  2  2  4  4  4  4  2  3  4  4  4  4  4  4  4  3  2  2  2  2  2  2  2  2  2  2  2  2  3  3  3  2  2  1  2  2  2  2  2  2  2  2  2  2  2  2  2  2  2  2  2  2  2  2  2  2  2  3  3  3  3  3  3  3  3  3  6  3  3  3  3  3  4  4  4  4  4  4  4  4  4  4  3  3  3  3  3  2  2  3  3  7  7  6  6  3  3  3  9  6  3  3  3  10  8  8  8  3  3  3  9  2  2  2  2  2  2  2  1  1  1 | Yoghurt | Mongolia | 2009 |
| IMAU20318 | ST5 | CC1 | 2 | 2 | 2 | 1 | 4 | 11 | 3 | 2 | 4 | 1 | 1  2  2  2  2  2  2  2  2  2  2  2  2  2  2  2  2  2  2  2  2  2  2  2  3  3  3  3  3  2  2  2  2  2  2  2  2  2  2  2  2  2  4  4  4  2  2  3  2  2  5  5  5  5  2  2  4  4  4  4  2  3  4  4  4  4  4  4  4  3  2  2  2  2  2  2  2  2  2  2  2  2  3  3  3  2  2  1  2  2  2  2  2  2  2  2  2  2  2  2  2  2  2  2  2  2  2  2  2  2  2  3  3  3  3  3  3  3  3  3  6  3  3  3  3  3  4  4  4  4  4  4  4  4  4  4  3  3  3  3  3  2  2  3  3  7  7  6  6  3  3  3  9  6  3  3  3  10  8  8  8  3  3  3  9  2  2  2  2  2  2  2  1  1  1 | Yoghurt | Mongolia | 2009 |
| IMAU20375 | ST5 | CC1 | 2 | 2 | 2 | 1 | 4 | 11 | 3 | 2 | 4 | 1 | 1  2  2  2  2  2  2  2  2  2  2  2  2  2  2  2  2  2  2  2  2  2  2  2  3  3  3  3  3  2  2  2  2  2  2  2  2  2  2  2  2  2  4  4  4  2  2  3  2  2  5  5  5  5  2  2  4  4  4  4  2  3  4  4  4  4  4  4  4  3  2  2  2  2  2  2  2  2  2  2  2  2  3  3  3  2  2  1  2  2  2  2  2  2  2  2  2  2  2  2  2  2  2  2  2  2  2  2  2  2  2  3  3  3  3  3  3  3  3  3  6  3  3  3  3  3  4  4  4  4  4  4  4  4  4  4  3  3  3  3  3  2  2  3  3  7  7  6  6  3  3  3  9  6  3  3  3  10  8  8  8  3  3  3  9  2  2  2  2  2  2  2  1  1  1 | Yoghurt | Mongolia | 2009 |
| IMAU20376 | ST5 | CC1 | 2 | 2 | 2 | 1 | 4 | 11 | 3 | 2 | 4 | 1 | 1  2  2  2  2  2  2  2  2  2  2  2  2  2  2  2  2  2  2  2  2  2  2  2  3  3  3  3  3  2  2  2  2  2  2  2  2  2  2  2  2  2  4  4  4  2  2  3  2  2  5  5  5  5  2  2  4  4  4  4  2  3  4  4  4  4  4  4  4  3  2  2  2  2  2  2  2  2  2  2  2  2  3  3  3  2  2  1  2  2  2  2  2  2  2  2  2  2  2  2  2  2  2  2  2  2  2  2  2  2  2  3  3  3  3  3  3  3  3  3  6  3  3  3  3  3  4  4  4  4  4  4  4  4  4  4  3  3  3  3  3  2  2  3  3  7  7  6  6  3  3  3  9  6  3  3  3  10  8  8  8  3  3  3  9  2  2  2  2  2  2  2  1  1  1 | Yoghurt | Mongolia | 2009 |
| IMAU20413 | ST5 | CC1 | 2 | 2 | 2 | 1 | 4 | 11 | 3 | 2 | 4 | 1 | 1  2  2  2  2  2  2  2  2  2  2  2  2  2  2  2  2  2  2  2  2  2  2  2  3  3  3  3  3  2  2  2  2  2  2  2  2  2  2  2  2  2  4  4  4  2  2  3  2  2  5  5  5  5  2  2  4  4  4  4  2  3  4  4  4  4  4  4  4  3  2  2  2  2  2  2  2  2  2  2  2  2  3  3  3  2  2  1  2  2  2  2  2  2  2  2  2  2  2  2  2  2  2  2  2  2  2  2  2  2  2  3  3  3  3  3  3  3  3  3  6  3  3  3  3  3  4  4  4  4  4  4  4  4  4  4  3  3  3  3  3  2  2  3  3  7  7  6  6  3  3  3  9  6  3  3  3  10  8  8  8  3  3  3  9  2  2  2  2  2  2  2  1  1  1 | Yoghurt | Mongolia | 2009 |
| IMAU20677 | ST5 | CC1 | 2 | 2 | 2 | 1 | 4 | 11 | 3 | 2 | 4 | 1 | 1  2  2  2  2  2  2  2  2  2  2  2  2  2  2  2  2  2  2  2  2  2  2  2  3  3  3  3  3  2  2  2  2  2  2  2  2  2  2  2  2  2  4  4  4  2  2  3  2  2  5  5  5  5  2  2  4  4  4  4  2  3  4  4  4  4  4  4  4  3  2  2  2  2  2  2  2  2  2  2  2  2  3  3  3  2  2  1  2  2  2  2  2  2  2  2  2  2  2  2  2  2  2  2  2  2  2  2  2  2  2  3  3  3  3  3  3  3  3  3  6  3  3  3  3  3  4  4  4  4  4  4  4  4  4  4  3  3  3  3  3  2  2  3  3  7  7  6  6  3  3  3  9  6  3  3  3  10  8  8  8  3  3  3  9  2  2  2  2  2  2  2  1  1  1 | Yoghurt | Mongolia | 2009 |
| IMAU20694 | ST5 | CC1 | 2 | 2 | 2 | 1 | 4 | 11 | 3 | 2 | 4 | 1 | 1  2  2  2  2  2  2  2  2  2  2  2  2  2  2  2  2  2  2  2  2  2  2  2  3  3  3  3  3  2  2  2  2  2  2  2  2  2  2  2  2  2  4  4  4  2  2  3  2  2  5  5  5  5  2  2  4  4  4  4  2  3  4  4  4  4  4  4  4  3  2  2  2  2  2  2  2  2  2  2  2  2  3  3  3  2  2  1  2  2  2  2  2  2  2  2  2  2  2  2  2  2  2  2  2  2  2  2  2  2  2  3  3  3  3  3  3  3  3  3  6  3  3  3  3  3  4  4  4  4  4  4  4  4  4  4  3  3  3  3  3  2  2  3  3  7  7  6  6  3  3  3  9  6  3  3  3  10  8  8  8  3  3  3  9  2  2  2  2  2  2  2  1  1  1 | Yoghurt | Mongolia | 2009 |
| IMAU20698 | ST5 | CC1 | 2 | 2 | 2 | 1 | 4 | 11 | 3 | 2 | 4 | 1 | 1  2  2  2  2  2  2  2  2  2  2  2  2  2  2  2  2  2  2  2  2  2  2  2  3  3  3  3  3  2  2  2  2  2  2  2  2  2  2  2  2  2  4  4  4  2  2  3  2  2  5  5  5  5  2  2  4  4  4  4  2  3  4  4  4  4  4  4  4  3  2  2  2  2  2  2  2  2  2  2  2  2  3  3  3  2  2  1  2  2  2  2  2  2  2  2  2  2  2  2  2  2  2  2  2  2  2  2  2  2  2  3  3  3  3  3  3  3  3  3  6  3  3  3  3  3  4  4  4  4  4  4  4  4  4  4  3  3  3  3  3  2  2  3  3  7  7  6  6  3  3  3  9  6  3  3  3  10  8  8  8  3  3  3  9  2  2  2  2  2  2  2  1  1  1 | Yoghurt | Mongolia | 2009 |
| IMAU20700 | ST5 | CC1 | 2 | 2 | 2 | 1 | 4 | 11 | 3 | 2 | 4 | 1 | 1  2  2  2  2  2  2  2  2  2  2  2  2  2  2  2  2  2  2  2  2  2  2  2  3  3  3  3  3  2  2  2  2  2  2  2  2  2  2  2  2  2  4  4  4  2  2  3  2  2  5  5  5  5  2  2  4  4  4  4  2  3  4  4  4  4  4  4  4  3  2  2  2  2  2  2  2  2  2  2  2  2  3  3  3  2  2  1  2  2  2  2  2  2  2  2  2  2  2  2  2  2  2  2  2  2  2  2  2  2  2  3  3  3  3  3  3  3  3  3  6  3  3  3  3  3  4  4  4  4  4  4  4  4  4  4  3  3  3  3  3  2  2  3  3  7  7  6  6  3  3  3  9  6  3  3  3  10  8  8  8  3  3  3  9  2  2  2  2  2  2  2  1  1  1 | Yoghurt | Mongolia | 2009 |
| IMAU20704 | ST5 | CC1 | 2 | 2 | 2 | 1 | 4 | 11 | 3 | 2 | 4 | 1 | 1  2  2  2  2  2  2  2  2  2  2  2  2  2  2  2  2  2  2  2  2  2  2  2  3  3  3  3  3  2  2  2  2  2  2  2  2  2  2  2  2  2  4  4  4  2  2  3  2  2  5  5  5  5  2  2  4  4  4  4  2  3  4  4  4  4  4  4  4  3  2  2  2  2  2  2  2  2  2  2  2  2  3  3  3  2  2  1  2  2  2  2  2  2  2  2  2  2  2  2  2  2  2  2  2  2  2  2  2  2  2  3  3  3  3  3  3  3  3  3  6  3  3  3  3  3  4  4  4  4  4  4  4  4  4  4  3  3  3  3  3  2  2  3  3  7  7  6  6  3  3  3  9  6  3  3  3  10  8  8  8  3  3  3  9  2  2  2  2  2  2  2  1  1  1 | Yoghurt | Mongolia | 2009 |
| IMAU20050 | ST6 | singleton | 9 | 1 | 2 | 4 | 10 | 2 | 2 | 3 | 2 | 5 | 2 | Fermented camels’ milk | Mongolia | 2006 |
| IMAU70041 | ST6 | singleton | 9 | 1 | 2 | 4 | 10 | 2 | 2 | 3 | 2 | 5 | 2 | Acidic gruel | Inner Mongolia | 2008 |
| IMAU70075 | ST6 | singleton | 9 | 1 | 2 | 4 | 10 | 2 | 2 | 3 | 2 | 5 | 2 | Acidic gruel | Inner Mongolia | 2008 |
| IMAU70076 | ST6 | singleton | 9 | 1 | 2 | 4 | 10 | 2 | 2 | 3 | 2 | 5 | 2 | Acidic gruel | Inner Mongolia | 2008 |
| IMAU70162 | ST6 | singleton | 9 | 1 | 2 | 4 | 10 | 2 | 2 | 3 | 2 | 5 | 2 | Acidic gruel | Inner Mongolia | 2008 |
| IMAU70166 | ST6 | singleton | 9 | 1 | 2 | 4 | 10 | 2 | 2 | 3 | 2 | 5 | 2 | Acidic gruel | Inner Mongolia | 2008 |
| IMAU20159 | ST7 | singleton | 7 | 11 | 2 | 1 | 4 | 11 | 1 | 2 | 4 | 9 | 1 | Yoghurt | Mongolia | 2009 |
| IMAU20219 | ST8 | singleton | 4 | 1 | 6 | 5 | 2 | 9 | 10 | 4 | 1 | 1 | 4 | Yoghurt | Mongolia | 2009 |
| IMAU20224 | ST8 | singleton | 4 | 1 | 6 | 5 | 2 | 9 | 10 | 4 | 1 | 1 | 4 | Yoghurt | Mongolia | 2009 |
| IMAU20225 | ST8 | singleton | 4 | 1 | 6 | 5 | 2 | 9 | 10 | 4 | 1 | 1 | 4 | Yoghurt | Mongolia | 2009 |
| IMAU20263 | ST9 | CC1 | 2 | 2 | 2 | 1 | 4 | 11 | 1 | 2 | 4 | 8 | 1 | Yoghurt | Mongolia | 2009 |
| IMAU20268 | ST9 | CC1 | 2 | 2 | 2 | 1 | 4 | 11 | 1 | 2 | 4 | 8 | 1 | Yoghurt | Mongolia | 2009 |
| IMAU20330 | ST10 | singleton | 3 | 3 | 2 | 1 | 4 | 2 | 1 | 3 | 4 | 1 | 1 | Yoghurt | Mongolia | 2009 |
| IMAU20436 | ST11 | CC1 | 2 | 2 | 2 | 1 | 4 | 11 | 1 | 2 | 5 | 1 | 1 | Yoghurt | Mongolia | 2009 |
| IMAU20522 | ST11 | CC1 | 2 | 2 | 2 | 1 | 4 | 11 | 1 | 2 | 5 | 1 | 1 | Yoghurt | Mongolia | 2009 |
| IMAU20523 | ST11 | CC1 | 2 | 2 | 2 | 1 | 4 | 11 | 1 | 2 | 5 | 1 | 1 | Yoghurt | Mongolia | 2009 |
| IMAU20439 | ST12 | CC1 | 2 | 2 | 2 | 1 | 4 | 11 | 1 | 5 | 4 | 1 | 1 | Yoghurt | Mongolia | 2009 |
| IMAU20440 | ST12 | CC1 | 2 | 2 | 2 | 1 | 4 | 11 | 1 | 5 | 4 | 1 | 1 | Yoghurt | Mongolia | 2009 |
| IMAU20442 | ST12 | CC1 | 2 | 2 | 2 | 1 | 4 | 11 | 1 | 5 | 4 | 1 | 1 | Yoghurt | Mongolia | 2009 |
| IMAU20443 | ST12 | CC1 | 2 | 2 | 2 | 1 | 4 | 11 | 1 | 5 | 4 | 1 | 1 | Yoghurt | Mongolia | 2009 |
| IMAU20675 | ST13 | singleton | 2 | 3 | 2 | 1 | 2 | 11 | 2 | 4 | 4 | 1 | 1 | Yoghurt | Mongolia | 2009 |
| IMAU20681 | ST14 | singleton | 3 | 3 | 2 | 1 | 2 | 11 | 1 | 4 | 4 | 1 | 1 | Yoghurt | Mongolia | 2009 |
| IMAU20715 | ST15 | singleton | 8 | 1 | 5 | 1 | 2 | 2 | 9 | 4 | 1 | 6 | 4 | Yoghurt | Mongolia | 2009 |
| IMAU20717 | ST15 | singleton | 8 | 1 | 5 | 1 | 2 | 2 | 9 | 4 | 1 | 6 | 4 | Yoghurt | Mongolia | 2009 |
| IMAU30108 | ST16 | CC1 | 2 | 7 | 2 | 1 | 4 | 11 | 4 | 2 | 4 | 1 | 1 | Koumiss | XinJiang | 2004 |
| IMAU40125 | ST17 | CC1 | 2 | 2 | 2 | 1 | 4 | 11 | 1 | 3 | 4 | 1 | 1 | Kurut | Qinghai | 2005 |
| IMAU50008 | ST18 | CC2 | 4 | 3 | 2 | 3 | 1 | 5 | 6 | 4 | 1 | 4 | 8 | Milk fan acid whey | Yunnan | 2006 |
| IMAU50071 | ST18 | CC2 | 4 | 3 | 2 | 3 | 1 | 5 | 6 | 4 | 1 | 4 | 8 | Milk fan acid whey | Yunnan | 2006 |
| IMAU50074 | ST18 | CC2 | 4 | 3 | 2 | 3 | 1 | 5 | 6 | 4 | 1 | 4 | 8 | Milk fan acid whey | Yunnan | 2006 |
| IMAU50077 | ST18 | CC2 | 4 | 3 | 2 | 3 | 1 | 5 | 6 | 4 | 1 | 4 | 8 | Milk fan acid whey | Yunnan | 2006 |
| IMAU50078 | ST18 | CC2 | 4 | 3 | 2 | 3 | 1 | 5 | 6 | 4 | 1 | 4 | 8 | Milk fan acid whey | Yunnan | 2006 |
| IMAU50087 | ST18 | CC2 | 4 | 3 | 2 | 3 | 1 | 5 | 6 | 4 | 1 | 4 | 8 | Milk fan acid whey | Yunnan | 2006 |
| IMAU50086 | ST19 | singleton | 4 | 7 | 2 | 3 | 1 | 11 | 6 | 4 | 1 | 6 | 2 | Milk fan acid whey | Yunnan | 2006 |
| IMAU50088 | ST20 | CC2 | 4 | 3 | 2 | 3 | 1 | 5 | 6 | 3 | 1 | 4 | 8 | Milk fan acid whey | Yunnan | 2006 |
| IMAU60072 | ST21 | CC1 | 2 | 2 | 2 | 1 | 4 | 10 | 1 | 2 | 4 | 1 | 1 | Kurut | Tibet | 2007 |
| IMAU60073 | ST21 | CC1 | 2 | 2 | 2 | 1 | 4 | 10 | 1 | 2 | 4 | 1 | 1 | Kurut | Tibet | 2007 |
| IMAU60075 | ST21 | CC1 | 2 | 2 | 2 | 1 | 4 | 10 | 1 | 2 | 4 | 1 | 1 | Kurut | Tibet | 2007 |
| IMAU60077 | ST21 | CC1 | 2 | 2 | 2 | 1 | 4 | 10 | 1 | 2 | 4 | 1 | 1 | Kurut | Tibet | 2007 |
| IMAU60078 | ST21 | CC1 | 2 | 2 | 2 | 1 | 4 | 10 | 1 | 2 | 4 | 1 | 1 | Kurut | Tibet | 2007 |
| IMAU60076 | ST22 | singleton | 3 | 1 | 4 | 1 | 8 | 2 | 2 | 3 | 3 | 3 | 2 | Kurut | Tibet | 2007 |
| IMAU70058 | ST22 | singleton | 3 | 1 | 4 | 1 | 8 | 2 | 2 | 3 | 3 | 3 | 2 | Acidic gruel | Inner Mongolia | 2008 |
| IMAU70059 | ST22 | singleton | 3 | 1 | 4 | 1 | 8 | 2 | 2 | 3 | 3 | 3 | 2 | Acidic gruel | Inner Mongolia | 2008 |
| IMAU60080 | ST23 | CC1 | 2 | 2 | 2 | 1 | 4 | 11 | 4 | 2 | 4 | 1 | 1 | Kurut | Tibet | 2007 |
| IMAU60083 | ST23 | CC1 | 2 | 2 | 2 | 1 | 4 | 11 | 4 | 2 | 4 | 1 | 1 | Kurut | Tibet | 2007 |
| IMAU60092 | ST23 | CC1 | 2 | 2 | 2 | 1 | 4 | 11 | 4 | 2 | 4 | 1 | 1 | Kurut | Tibet | 2007 |
| IMAU60142 | ST23 | CC1 | 2 | 2 | 2 | 1 | 4 | 11 | 4 | 2 | 4 | 1 | 1 | Kurut | Tibet | 2007 |
| IMAU60152 | ST23 | CC1 | 2 | 2 | 2 | 1 | 4 | 11 | 4 | 2 | 4 | 1 | 1 | Kurut | Tibet | 2007 |
| IMAU60154 | ST23 | CC1 | 2 | 2 | 2 | 1 | 4 | 11 | 4 | 2 | 4 | 1 | 1 | Kurut | Tibet | 2007 |
| IMAU60155 | ST23 | CC1 | 2 | 2 | 2 | 1 | 4 | 11 | 4 | 2 | 4 | 1 | 1 | Kurut | Tibet | 2007 |
| IMAU60162 | ST23 | CC1 | 2 | 2 | 2 | 1 | 4 | 11 | 4 | 2 | 4 | 1 | 1 | Kurut | Tibet | 2007 |
| IMAU60164 | ST23 | CC1 | 2 | 2 | 2 | 1 | 4 | 11 | 4 | 2 | 4 | 1 | 1 | Kurut | Tibet | 2007 |
| IMAU60168 | ST23 | CC1 | 2 | 2 | 2 | 1 | 4 | 11 | 4 | 2 | 4 | 1 | 1 | Kurut | Tibet | 2007 |
| IMAU60085 | ST24 | CC1 | 2 | 2 | 2 | 1 | 4 | 11 | 18 | 2 | 4 | 1 | 1 | Kurut | Tibet | 2007 |
| IMAU60087 | ST24 | CC1 | 2 | 2 | 2 | 1 | 4 | 11 | 18 | 2 | 4 | 1 | 1 | Kurut | Tibet | 2007 |
| IMAU60090 | ST24 | CC1 | 2 | 2 | 2 | 1 | 4 | 11 | 18 | 2 | 4 | 1 | 1 | Kurut | Tibet | 2007 |
| IMAU60111 | ST25 | CC1 | 5 | 2 | 2 | 1 | 4 | 11 | 1 | 2 | 4 | 1 | 1 | Kurut | Tibet | 2007 |
| IMAU60157 | ST25 | CC1 | 5 | 2 | 2 | 1 | 4 | 11 | 1 | 2 | 4 | 1 | 1 | Kurut | Tibet | 2007 |
| IMAU60112 | ST26 | CC1 | 1 | 2 | 2 | 1 | 4 | 11 | 4 | 2 | 4 | 1 | 1 | Kurut | Tibet | 2007 |
| IMAU60114 | ST26 | CC1 | 1 | 2 | 2 | 1 | 4 | 11 | 4 | 2 | 4 | 1 | 1 | Kurut | Tibet | 2007 |
| IMAU60115 | ST26 | CC1 | 1 | 2 | 2 | 1 | 4 | 11 | 4 | 2 | 4 | 1 | 1 | Kurut | Tibet | 2007 |
| IMAU60119 | ST26 | CC1 | 1 | 2 | 2 | 1 | 4 | 11 | 4 | 2 | 4 | 1 | 1 | Kurut | Tibet | 2007 |
| IMAU60137 | ST26 | CC1 | 1 | 2 | 2 | 1 | 4 | 11 | 4 | 2 | 4 | 1 | 1 | Kurut | Tibet | 2007 |
| IMAU60113 | ST27 | CC1 | 2 | 2 | 2 | 1 | 4 | 11 | 19 | 2 | 4 | 1 | 1 | Kurut | Tibet | 2007 |
| IMAU60144 | ST28 | CC1 | 5 | 2 | 2 | 9 | 4 | 11 | 1 | 2 | 4 | 1 | 1 | Kurut | Tibet | 2007 |
| IMAU60145 | ST28 | CC1 | 5 | 2 | 2 | 9 | 4 | 11 | 1 | 2 | 4 | 1 | 1 | Kurut | Tibet | 2007 |
| IMAU60147 | ST28 | CC1 | 5 | 2 | 2 | 9 | 4 | 11 | 1 | 2 | 4 | 1 | 1 | Kurut | Tibet | 2007 |
| IMAU60167 | ST29 | CC5 | 3 | 6 | 2 | 1 | 7 | 6 | 2 | 3 | 11 | 2 | 2 | Kurut | Tibet | 2007 |
| IMAU70055 | ST29 | CC5 | 3 | 6 | 2 | 1 | 7 | 6 | 2 | 3 | 11 | 2 | 2 | Acidic gruel | Inner Mongolia | 2008 |
| IMAU70064 | ST29 | CC5 | 3 | 6 | 2 | 1 | 7 | 6 | 2 | 3 | 11 | 2 | 2 | Acidic gruel | Inner Mongolia | 2008 |
| IMAU70065 | ST29 | CC5 | 3 | 6 | 2 | 1 | 7 | 6 | 2 | 3 | 11 | 2 | 2 | Acidic gruel | Inner Mongolia | 2008 |
| IMAU70003 | ST30 | singleton | 4 | 2 | 2 | 1 | 2 | 11 | 1 | 3 | 4 | 1 | 3 | Acidic gruel | Inner Mongolia | 2008 |
| IMAU70006 | ST31 | CC3 | 3 | 1 | 4 | 1 | 2 | 4 | 2 | 3 | 3 | 3 | 3 | Acidic gruel | Inner Mongolia | 2008 |
| IMAU70133 | ST31 | CC3 | 3 | 1 | 4 | 1 | 2 | 4 | 2 | 3 | 3 | 3 | 3 | Acidic gruel | Inner Mongolia | 2008 |
| IMAU70135 | ST31 | CC3 | 3 | 1 | 4 | 1 | 2 | 4 | 2 | 3 | 3 | 3 | 3 | Acidic gruel | Inner Mongolia | 2008 |
| IMAU70008 | ST32 | singleton | 3 | 3 | 2 | 11 | 10 | 2 | 14 | 3 | 2 | 2 | 7 | Acidic gruel | Inner Mongolia | 2008 |
| IMAU70013 | ST33 | singleton | 3 | 8 | 3 | 1 | 6 | 2 | 7 | 6 | 1 | 4 | 3 | Acidic gruel | Inner Mongolia | 2008 |
| IMAU70016 | ST34 | CC4 | 3 | 1 | 2 | 2 | 10 | 7 | 7 | 3 | 2 | 3 | 3 | Acidic gruel | Inner Mongolia | 2008 |
| IMAU70052 | ST34 | CC4 | 3 | 1 | 2 | 2 | 10 | 7 | 7 | 3 | 2 | 3 | 3 | Acidic gruel | Inner Mongolia | 2008 |
| IMAU70053 | ST34 | CC4 | 3 | 1 | 2 | 2 | 10 | 7 | 7 | 3 | 2 | 3 | 3 | Acidic gruel | Inner Mongolia | 2008 |
| IMAU70155 | ST34 | CC4 | 3 | 1 | 2 | 2 | 10 | 7 | 7 | 3 | 2 | 3 | 3 | Acidic gruel | Inner Mongolia | 2008 |
| IMAU70156 | ST34 | CC4 | 3 | 1 | 2 | 2 | 10 | 7 | 7 | 3 | 2 | 3 | 3 | Acidic gruel | Inner Mongolia | 2008 |
| IMAU70021 | ST35 | singleton | 3 | 2 | 2 | 1 | 4 | 11 | 6 | 4 | 4 | 1 | 1 | Acidic gruel | Inner Mongolia | 2008 |
| IMAU70031 | ST36 | singleton | 8 | 7 | 2 | 6 | 2 | 2 | 7 | 4 | 1 | 1 | 2 | Acidic gruel | Inner Mongolia | 2008 |
| IMAU70032 | ST37 | singleton | 3 | 4 | 2 | 1 | 2 | 2 | 5 | 4 | 2 | 2 | 3 | Acidic gruel | Inner Mongolia | 2008 |
| IMAU70145 | ST37 | singleton | 3 | 4 | 2 | 1 | 2 | 2 | 5 | 4 | 2 | 2 | 3 | Acidic gruel | Inner Mongolia | 2008 |
| IMAU70146 | ST37 | singleton | 3 | 4 | 2 | 1 | 2 | 2 | 5 | 4 | 2 | 2 | 3 | Acidic gruel | Inner Mongolia | 2008 |
| IMAU70147 | ST37 | singleton | 3 | 4 | 2 | 1 | 2 | 2 | 5 | 4 | 2 | 2 | 3 | Acidic gruel | Inner Mongolia | 2008 |
| IMAU70148 | ST37 | singleton | 3 | 4 | 2 | 1 | 2 | 2 | 5 | 4 | 2 | 2 | 3 | Acidic gruel | Inner Mongolia | 2008 |
| IMAU70149 | ST37 | singleton | 3 | 4 | 2 | 1 | 2 | 2 | 5 | 4 | 2 | 2 | 3 | Acidic gruel | Inner Mongolia | 2008 |
| IMAU70150 | ST37 | singleton | 3 | 4 | 2 | 1 | 2 | 2 | 5 | 4 | 2 | 2 | 3 | Acidic gruel | Inner Mongolia | 2008 |
| IMAU70151 | ST37 | singleton | 3 | 4 | 2 | 1 | 2 | 2 | 5 | 4 | 2 | 2 | 3 | Acidic gruel | Inner Mongolia | 2008 |
| IMAU70047 | ST38 | CC3 | 3 | 1 | 4 | 1 | 2 | 4 | 8 | 3 | 3 | 3 | 3 | Acidic gruel | Inner Mongolia | 2008 |
| IMAU70152 | ST38 | CC3 | 3 | 1 | 4 | 1 | 2 | 4 | 8 | 3 | 3 | 3 | 3 | Acidic gruel | Inner Mongolia | 2008 |
| IMAU70153 | ST38 | CC3 | 3 | 1 | 4 | 1 | 2 | 4 | 8 | 3 | 3 | 3 | 3 | Acidic gruel | Inner Mongolia | 2008 |
| IMAU70154 | ST38 | CC3 | 3 | 1 | 4 | 1 | 2 | 4 | 8 | 3 | 3 | 3 | 3 | Acidic gruel | Inner Mongolia | 2008 |
| IMAU70050 | ST39 | CC4 | 3 | 1 | 2 | 2 | 10 | 8 | 7 | 3 | 2 | 3 | 3 | Acidic gruel | Inner Mongolia | 2008 |
| IMAU70051 | ST40 | singleton | 8 | 1 | 2 | 8 | 2 | 2 | 11 | 2 | 1 | 6 | 3 | Acidic gruel | Inner Mongolia | 2008 |
| IMAU70086 | ST40 | singleton | 8 | 1 | 2 | 8 | 2 | 2 | 11 | 2 | 1 | 6 | 3 | Acidic gruel | Inner Mongolia | 2008 |
| IMAU70054 | ST41 | singleton | 8 | 1 | 2 | 1 | 7 | 2 | 5 | 3 | 9 | 3 | 6 | Acidic gruel | Inner Mongolia | 2008 |
| IMAU70056 | ST42 | CC1 | 4 | 2 | 2 | 1 | 4 | 11 | 1 | 3 | 4 | 1 | 1 | Acidic gruel | Inner Mongolia | 2008 |
| IMAU70069 | ST43 | singleton | 8 | 3 | 7 | 1 | 5 | 1 | 13 | 7 | 1 | 4 | 2 | Acidic gruel | Inner Mongolia | 2008 |
| IMAU70077 | ST43 | singleton | 8 | 3 | 7 | 1 | 5 | 1 | 13 | 7 | 1 | 4 | 2 | Acidic gruel | Inner Mongolia | 2008 |
| IMAU70132 | ST44 | singleton | 3 | 5 | 7 | 1 | 9 | 2 | 15 | 6 | 7 | 2 | 3 | Acidic gruel | Inner Mongolia | 2008 |
| IMAU70134 | ST44 | singleton | 3 | 5 | 7 | 1 | 9 | 2 | 15 | 6 | 7 | 2 | 3 | Acidic gruel | Inner Mongolia | 2008 |
| IMAU70136 | ST45 | singleton | 3 | 3 | 2 | 1 | 10 | 2 | 2 | 3 | 8 | 3 | 2 | Acidic gruel | Inner Mongolia | 2008 |
| IMAU70137 | ST45 | singleton | 3 | 3 | 2 | 1 | 10 | 2 | 2 | 3 | 8 | 3 | 2 | Acidic gruel | Inner Mongolia | 2008 |
| IMAU70138 | ST45 | singleton | 3 | 3 | 2 | 1 | 10 | 2 | 2 | 3 | 8 | 3 | 2 | Acidic gruel | Inner Mongolia | 2008 |
| IMAU70139 | ST46 | singleton | 10 | 1 | 2 | 5 | 2 | 2 | 7 | 9 | 2 | 11 | 2 | Acidic gruel | Inner Mongolia | 2008 |
| IMAU70140 | ST47 | singleton | 3 | 3 | 3 | 12 | 6 | 2 | 7 | 6 | 1 | 4 | 3 | Acidic gruel | Inner Mongolia | 2008 |
| IMAU70141 | ST48 | singleton | 3 | 3 | 2 | 2 | 10 | 2 | 2 | 3 | 2 | 2 | 2 | Acidic gruel | Inner Mongolia | 2008 |
| IMAU70143 | ST48 | singleton | 3 | 3 | 2 | 2 | 10 | 2 | 2 | 3 | 2 | 2 | 2 | Acidic gruel | Inner Mongolia | 2008 |
| IMAU70144 | ST48 | singleton | 3 | 3 | 2 | 2 | 10 | 2 | 2 | 3 | 2 | 2 | 2 | Acidic gruel | Inner Mongolia | 2008 |
| IMAU70142 | ST49 | singleton | 3 | 3 | 7 | 1 | 5 | 2 | 2 | 10 | 2 | 12 | 2 | Acidic gruel | Inner Mongolia | 2008 |
| IMAU70157 | ST50 | singleton | 3 | 5 | 3 | 7 | 5 | 2 | 15 | 8 | 2 | 1 | 5 | Acidic gruel | Inner Mongolia | 2008 |
| IMAU70158 | ST50 | singleton | 3 | 5 | 3 | 7 | 5 | 2 | 15 | 8 | 2 | 1 | 5 | Acidic gruel | Inner Mongolia | 2008 |
| IMAU70159 | ST50 | singleton | 3 | 5 | 3 | 7 | 5 | 2 | 15 | 8 | 2 | 1 | 5 | Acidic gruel | Inner Mongolia | 2008 |
| IMAU70160 | ST51 | CC5 | 3 | 6 | 2 | 1 | 7 | 6 | 7 | 3 | 11 | 2 | 2 | Acidic gruel | Inner Mongolia | 2008 |
| IMAU70161 | ST52 | singleton | 3 | 1 | 2 | 2 | 5 | 2 | 17 | 3 | 2 | 2 | 2 | Acidic gruel | Inner Mongolia | 2008 |
| IMAU70167 | ST53 | singleton | 3 | 9 | 2 | 6 | 2 | 2 | 16 | 9 | 1 | 4 | 2 | Acidic gruel | Inner Mongolia | 2008 |
| IMAU80232 | ST54 | singleton | 2 | 10 | 2 | 1 | 4 | 11 | 1 | 2 | 4 | 10 | 1 | Kurut | Sichuan | 2009 |
| IMAU80361 | ST55 | singleton | 2 | 2 | 1 | 1 | 4 | 11 | 1 | 2 | 6 | 7 | 1 | Qula | Sichuan | 2009 |
| IMAU80363 | ST55 | singleton | 2 | 2 | 1 | 1 | 4 | 11 | 1 | 2 | 6 | 7 | 1 | Qula | Sichuan | 2009 |
| IMAU80374 | ST55 | singleton | 2 | 2 | 1 | 1 | 4 | 11 | 1 | 2 | 6 | 7 | 1 | Whey | Sichuan | 2009 |
| IMAU80800 | ST55 | singleton | 2 | 2 | 1 | 1 | 4 | 11 | 1 | 2 | 6 | 7 | 1 | Kurut | Gansu | 2009 |
| IMAU80814 | ST55 | singleton | 2 | 2 | 1 | 1 | 4 | 11 | 1 | 2 | 6 | 7 | 1 | Whey | Gansu | 2009 |
| IMAU80464 | ST56 | singleton | 6 | 2 | 2 | 1 | 4 | 11 | 1 | 1 | 1 | 1 | 9 | Qula | Sichuan | 2009 |
| IMAU80778 | ST56 | singleton | 6 | 2 | 2 | 1 | 4 | 11 | 1 | 1 | 1 | 1 | 9 | Kurut | Gansu | 2009 |
| IMAU80780 | ST56 | singleton | 6 | 2 | 2 | 1 | 4 | 11 | 1 | 1 | 1 | 1 | 9 | Kurut | Gansu | 2009 |
| IFO3956 | ST57 | singleton | 8 | 1 | 2 | 1 | 11 | 2 | 12 | 3 | 12 | 1 | 3 | Fermented plant material | Japan | - |
