# Supplementary figures and images for: The evolution and population structure of Lactobacillus fermentum from different naturally fermented products as determined by multilocus sequence typing (MLST)
Source: BMC Microbiol. 2015 May 20;15:107. doi: 10.1186/s12866-015-0447-z (PMC4437502; doi:10.1186/s12866-015-0447-z)

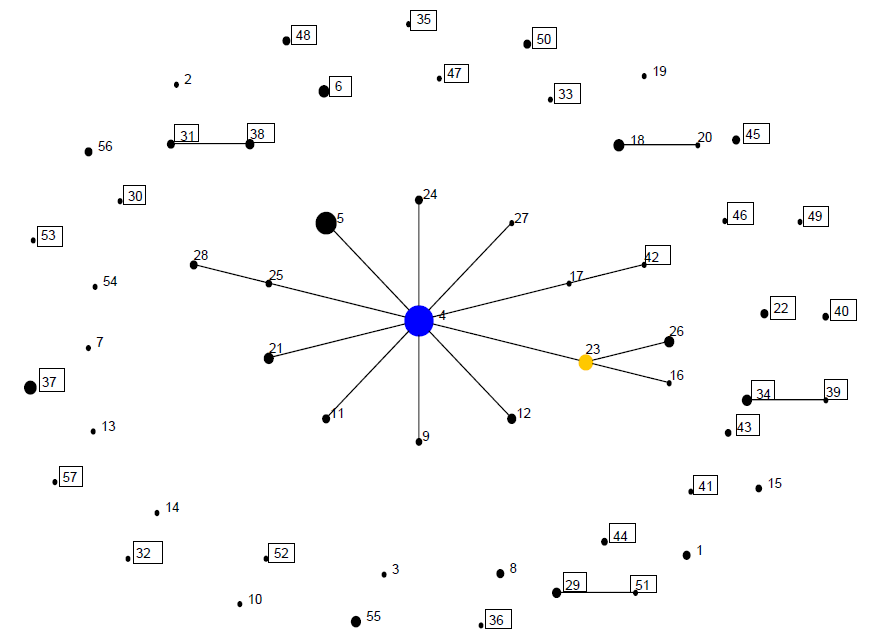

Supplement: Additional file 2: Figure S1. — eBURST analysis of 203 L. fermentum isolates used in this study. In the eBURST diagram, five clonal and thirty-four singletons are shown. The primary founders of eBURST groups are positioned centrally in the cluster and labeled in blue, and the subgroup founders are shown in yellow. Dots indicate sequence type and lines connect single-locus variants, which are STs that differ in only one of the 11 housekeeping gene fragments. Boxed numbers indicate STs found in acidic gruel, the other numbers indicated STs found in dairy products. [file 12866_2015_447_MOESM2_ESM.png]
